# Gene regulatory networking reveals the molecular cue to lysophosphatidic acid‐induced metabolic adaptations in ovarian cancer cells

**DOI:** 10.1002/1878-0261.12046

**Published:** 2017-04-03

**Authors:** Upasana Ray, Shreya Roy Chowdhury, Madavan Vasudevan, Kiran Bankar, Susanta Roychoudhury, Sib Sankar Roy

**Affiliations:** ^1^ Cell Biology and Physiology Division CSIR‐Indian Institute of Chemical Biology Council of Scientific and Industrial Research Kolkata India; ^2^ Bionivid Technology Private Limited Bangalore India; ^3^ Saroj Gupta Cancer Centre and Research Institute Kolkata India

**Keywords:** ETS‐1, HIF‐1α, invasion, lysophosphatidic acid, metabolic adaptations, ovarian cancer

## Abstract

Extravasation and metastatic progression are two main reasons for the high mortality rate associated with cancer. The metastatic potential of cancer cells depends on a plethora of metabolic challenges prevailing within the tumor microenvironment. To achieve higher rates of proliferation, cancer cells reprogram their metabolism, increasing glycolysis and biosynthetic activities. Just why this metabolic reprogramming predisposes cells towards increased oncogenesis remains elusive. The accumulation of myriad oncolipids in the tumor microenvironment has been shown to promote the invasiveness of cancer cells, with lysophosphatidic acid (LPA) being one such critical factor enriched in ovarian cancer patients. Cellular bioenergetic studies confirm that oxidative phosphorylation is suppressed and glycolysis is increased with long exposure to LPA in ovarian cancer cells compared with non‐transformed epithelial cells. We sought to uncover the regulatory complexity underlying this oncolipid‐induced metabolic perturbation. Gene regulatory networking using RNA‐Seq analysis identified the oncogene *ETS‐1* as a critical mediator of LPA‐induced metabolic alterations for the maintenance of invasive phenotype. Moreover, LPA receptor‐2 specific PtdIns3K‐AKT signaling induces ETS‐1 and its target matrix metalloproteases. Abrogation of ETS‐1 restores cellular bioenergetics towards increased oxidative phosphorylation and reduced glycolysis, and this effect was reversed by the presence of LPA. Furthermore, the bioenergetic status of LPA‐treated ovarian cancer cells mimics hypoxia through induction of hypoxia‐inducible factor‐1α, which was found to transactivate *ets‐1*. Studies in primary tumors generated in syngeneic mice corroborated the *in vitro* findings. Thus, our study highlights the phenotypic changes induced by the pro‐metastatic factor ETS‐1 in ovarian cancer cells. The relationship between enhanced invasiveness and metabolic plasticity further illustrates the critical role of metabolic adaptation of cancer cells as a driver of tumor progression. These findings reveal oncolipid‐induced metabolic predisposition as a new mechanism of tumorigenesis and propose metabolic inhibitors as a potential approach for future management of aggressive ovarian cancer.

**Abbreviations**
AKT: protein kinase B
ChIP: chromatin immunoprecipitation
DAPI: 4′,6‐diamidino‐2‐phenylindole
ECAR: extracellular acidification rate
EMT: epithelial–mesenchymal transition
GAPDH: glyceraldehyde‐3 phosphate dehydrogenase
GSEA: gene set enrichment analysis
HIF: hypoxia inducible factor
IOSE: immortalized ovarian surface epithelium
LDHA: lactate dehydrogenase A
LPA: lysophosphatidic acid
LPAR: lysophosphatidic acid receptors
MAPK: mitogen‐activated protein kinase
MMP: matrix metalloprotease
OCR: oxygen consumption rate
PtdIns3K: phosphatidyl inositol 3‐kinase
PTX: pertussis toxin
RhoA: Ras homolog gene family, member A
siRNA: short interfering RNA
TGF‐β: transforming growth factor β
VEGF: vascular endothelial growth factor

## 1 Introduction

Most epithelial ovarian cancer patients present late‐stage metastasis, often with significant peritoneal ascites. The highly metastatic nature of the disease, and a lack of effective early detection, contribute to the low survival rate in ovarian cancer patients (Siegel *et al*., 2014). Consequently, a detailed understanding of the disease might lead to new therapeutic strategies and reduce the associated mortality rate.

The tumor microenvironment plays a critical role in determining the aggressiveness and malignancy of cancer (Xing *et al*., 2015). To sustain their rapid proliferation, most cancer cells redirect their energy sources toward aerobic glycolysis, a process termed the Warburg effect. This metabolic plasticity has emerged as a fundamental feature of transformed cells, promoting their metastatic potential (Hanahan and Weinberg, 2011); however, this process remains uncharacterized.

Lipid signaling through G‐protein coupled receptors has added a new dimension to signaling research (Birgbauer and Chun, 2006; Xu *et al*., 2006). G‐protein coupled receptors are characterized by their ability to classify and respond to chemically diverse ligands (Dorsam and Gutkind, 2007). Lysophospholipids, a ligand of G‐protein coupled receptors, perform their biological function through activation of G‐proteins coupled to a large family of cell‐surface receptors (Fang *et al*., 2000). Within this context, lysophosphatidic acid (LPA) has emerged as a critical tumor‐promoting metabolite that regulates diverse cellular responses (Mills and Moolenaar, 2003; Sengupta *et al*., 2004). LPA has been detected at significantly higher levels (1–80 μm) in the ascitic fluid of ovarian cancer patients, making it a potential biomarker for ovarian cancer (Xu *et al*., 1998, 2003). LPA is produced by hydrolysis of lyso‐phosphatidylcholine via membrane‐bound autotaxin activity; thereby generating an autocrine signaling loop through binding to its receptors (Hama *et al*., 2004; Sengupta *et al*., 2003). The major biological function of LPA is mediated through LPA_1_/EDG‐2, LPA_2_/EDG‐4 and LPA_3_/EDG‐7 receptors, depending on the cell type (Liu *et al*., 2009; Noguchi *et al*., 2009; Yu *et al*., 2008). However, most ovarian cancer cells have high levels of EDG‐4/EDG‐7 expression compared with non‐transformed ovarian surface epithelial cells (Fukushima *et al*., 2015; Murph *et al*., 2008). Previous studies have reported that LPA enhances proliferation and metastasis in numerous cancer cells (Fan *et al*., 2015; Fishman *et al*., 2001; Komachi *et al*., 2012; Leve *et al*., 2015; Park *et al*., 2011; So *et al*., 2004). Multiple intracellular signaling molecules such as mitogen‐activated protein kinases (MAPKs), Ras homolog gene family, member A (RhoA) and phosphatidyl inositol 3‐kinase (PtdIns3K) are activated by LPA, thereby linking the altered extracellular stimuli to varied cellular changes that support tumor progression (Bian *et al*., 2006; Jeong *et al*., 2012; Sautin *et al*., 2001). According to existing reports, metabolic reprogramming of cancer cells is a critical requirement for tumor progression. In this study, we investigate the role of LPA‐activated signaling in bioenergetic reprogramming to potentiate ovarian cancer progression.

Considering the critical involvement of LPA in promoting oncogenesis in various cancer types, we were prompted to investigate its potency towards metabolic modulation in ovarian cancer cells. Complex regulatory pathways were further uncovered to identify the molecular targets controlling the increased invasive potential of LPA‐treated cancer cells through transcriptome profiling.

## 2 Materials and methods

### 2.1 Cell culture and treatment

Human ovarian cancer cell lines SKOV‐3, PA‐1 (ATCC, Manassas, VA, USA) and OAW‐42 (Sigma‐Aldrich, St. Louis, MO, USA) were maintained in RPMI, minimal essential medium and Dulbecco's modified Eagle's medium, respectively; supplemented with 10% FBS, 100 μg·mL^−1^ streptomycin and 100 U·mL^−1^ penicillin (Invitrogen, Carlsbad, CA, USA). Human immortalized ovarian surface epithelial cells (IOSE‐364 from N. Aueresperg and C. Salamanca, Vancouver, Canada) were maintained in MCDB‐105 (Sigma‐Aldrich) and Medium‐199 (Invitrogen) in a 1 : 1 ratio supplemented as above. Human ovarian surface epithelial cells were isolated by scraping the surface of human ovarian tissue and immortalized by transfecting with SV40 large‐T antigen viral particles (Kruk *et al*., 1990). Murine ID8 ovarian cancer cells, were obtained from K. Roby (University of Kansas Medical Center, USA) and were maintained in Dulbecco's modified Eagle's medium supplemented with 4% FBS, 5 μg·mL^−1^ insulin, 5 μg·mL^−1^ transferrin and 5 ng·mL^−1^ sodium selenite (Sigma‐Aldrich).

LPA (Sigma) was used at a concentration of 20 μm unless otherwise specified. CoCl_2_ (Merck Millipore, Billerica, MA, USA, 100 μm) was used as hypoxia‐mimetic agent. Human recombinant transforming growth factor β (TGF‐β1; Calbiochem) at a concentration of 1 ng·mL^−1^ was used as a positive inducer of epithelial–mesenchymal transition (EMT). LPA‐receptor inhibitor [pertussis toxin (PTX), 100 nM], Akt inhibitor III (10 μm), PtdIns3K inhibitor (wortmanin, 100 nM), p38–MAPK inhibitor (SB203580, 10 μm), MEK inhibitor (U0126, 10 μm), JNK inhibitor II (20 μm) and hypoxia inducible factor (HIF)1α/2α inhibitor IV (300 nM) were from Calbiochem. HA‐HIF1alpha‐pcDNA3 was from Addgene. Prior to each treatment, cells were serum starved for 16 h, followed by 1 h treatment with inhibitors and then induced with LPA for 6 (mRNA) and 24 h (protein), unless otherwise specified.

### 2.2 Short interfering RNA and transfection

Short interfering RNAs (siRNAs) against ETS‐1, HIF‐1α, matrix metalloprotease MMP‐2, MMP‐9, MMP‐13, lysophosphatidic acid receptors LPAR‐1, LPAR‐2, LPAR‐3 (pooled siRNAs to nullify the off‐target effect, Santa Cruz Biotechnology, Dallas, TX, USA), and scrambled siRNA (Ambion) were used at a concentration of 20 nM following the protocol described earlier (Basu *et al*., 2014).

### 2.3 Quantitative real‐time PCR

Total RNAs were isolated using TRI‐Reagent (Sigma‐Aldrich) following the standard protocol (Basu and Roy, 2013). cDNA was synthesized using iScript (BioRad), following the manufacturer's instructions, followed by quantitative PCR (Basu *et al*., 2015). The primer sequences are given in Table 1.

**Table 1:** Sequence of the oligonucleotide primers used in quantitative PCR

Gene product	Forward primer (5′–3′)	Reverse primer (5′–3′)	Tm (°C)
*Hs_18s rRNA*	GATTCCGTGGGTGGTGGTGC	AAGAAGTTGGGGGACGCCGA	60
*Hs_ETS‐1*	GGAGGACCAGTCGTGGTAAA	AACTGCCATAGCTGGATTGG	59
*Hs_ETS‐2*	CAGCCACCGTCCCGACCAAG	GCTGGCTGGCGCTTGAGTGT	59
*Hs_MMP2*	TGATCTTGACCAGAATACCATCGA	GGCTTGCGAGGGAAGAAGTT	60
*Hs_MMP9*	ACCTCGAACTTTGACAGCGAC	GAGGAATGATCTAAGCCCAGC	60
*Hs_MMP13*	ATGACTGAGGCTCCGAGA	ACCTAAGGAGTGGCCGAACT	60
*Hs_HIF‐1α*	TGAACATAAAGTCTGCAACATGGA	TGAGGTTGGTTACTGTTGGTATCATATA	60
*Hs_CDH1*	GTCACTGACACCAACGATAATCT	TTTCAGTGTGGTGATTACGACGTA	60
*Hs_CLDN7*	GTGGCAGATGAGCTCCTATGC	CATCCACAGCCCCTTGTACA	60
*Hs_CDH2*	CCATCAAGCCTGTGGGAATC	GCAGATCGGACCGGATACTG	60
*Hs_FN1*	CCTTCATGGCAGCGGTTT	AGCGTCCTAAAGACTCCATGATCT	60
*Hs_LDHA*	GGTTCACAAGCAGGTTGGTTGA	CCAAATCTGCTACAGAGAGTCCAA	59
*HS_HK2*	GGGCATCTTTGAAACCAAGTTC	GGTGCTCTCAAGCCCTAAGTGT	61
*HS_PKM2*	CCGCCTGGACATTGATTCAC	GAAGCTGGGCCAATGGTACA	59

### 2.4 Western blot analysis

Cell lysis followed by protein extraction was performed as described previously (Basu *et al*., 2014). Lysates were subjected to immunoblotting with antibodies specific for ETS‐1, ETS‐2, MMP‐9, MMP‐13, HIF‐1α, vascular endothelial growth factor (VEGF), lactate dehydrogenase A (LDHA), glyceraldehyde‐3 phosphate dehydrogenase (GAPDH) (Santa Cruz Biotechnology; 1:2000); MMP‐2, protein kinase B (AKT), p‐AKT, proliferating cell nuclear antigen, E‐cadherin, N‐cadherin and vimentine (Cell Signaling, Danvers, MA, USA; 1 : 1000). Bands were visualized by reacting horseradish peroxidase‐labeled secondary antibodies (Cell signaling; 1 : 5000) with the ECL substrate (BioRad) by chemiluminesence.

### 2.5 Cellular respiration

Oxygen consumption rate (OCR) and extracellular acidification rate (ECAR) were measured using Seahorse XF‐24 Extracellular Flux Analyzer following the manufacturer's protocol. Briefly, ovarian cancer cells were seeded at 10 000 cells/24‐well plate (minimum of triplicate for each treatment), serum starved and treated with or without LPA or CoCl_2_ for 24 h. For ETS‐1 knockdown, cells were transiently transfected, serum starved for an additional 6 h, trypsinized, counted and equal numbers of cells were plated on a Seahorse plate in the presence or absence of LPA for 22 h. OCR measurement was performed with a XF Cell Mito‐Stress kit using the following inhibitors: oligomycin (1 μm), p‐trifluoromethoxy carbonyl cyanide phenyl hydrazone (FCCP, 1 μm) and rotenone/antimycin A mix (1 μm) sequentially in each well. ECAR was measured using a XF Glycolysis‐Stress kit in glucose‐free media with the following reagents: glucose (10 mm), oligomycin (1 μm) and 2‐deoxyglucose (25 mm) sequentially in each well. After all measurements, cells were lysed, the protein concentration was estimated to normalize the data, and data were analyzed by xf software.

### 2.6 Lactate production

Lactate levels in the conditioned media upon LPA treatment for 24 h were measured using a lactate assay kit (Sigma‐Aldrich) following the manufacturer's instructions. The results were provided as ‘fold change’ with respect to the control with significance.

### 2.7 *In vitro* invasion assay

*In vitro* cell invasion was studied using a Matrigel^®^ Invasion Chamber (BD Biosciences, San Jose, CA, USA) following the protocol described previously (Ghosh *et al*., 2012). Briefly 2.5 × 10^5^ cells per well were seeded in serum‐free medium in the upper chamber, and complete medium was added in the lower chamber. Cells were treated with inhibitors for 1 h followed by LPA induction for 22 h. To check the effect of silencing of ETS‐1, HIF‐1α, MMP‐2, MMP‐9 and MMP‐13, the cells were transiently transfected with the respective siRNAs on the day before plating in the Matrigel Invasion Chamber, followed by serum starvation for 16 h. The cells were subsequently trypsinized, counted, and equal numbers were placed in the upper chamber and allowed to invade in the presence and absence of LPA. Three separate experiments were performed followed by statistical analysis. Percent invasion was calculated as (number of cells invaded in treated/number of cells invaded in control) × 100.

### 2.8 Wound healing assay

Transfected cells were scratched with a 10 μL pipette tip, and supplied with new medium with or without LPA. For treatment, cells were starved, scratched and then exposed to inhibitors for 1 h followed by LPA. Images were taken at 0, 24 and 48 h using an inverted microscope (EVOS, Thermo Fisher Scientific).

### 2.9 MMP‐2 and MMP‐9 activity assay by gelatin zymography

After 24–48 h of treatment and transfections, culture supernatants (condition medium) were collected and concentrated. The concentrated condition medium was then subjected to 10% SDS/PAGE co‐polymerized with 0.1% gelatin, as described by Ghosh *et al*. (2012). Gels were stained with 0.1% Coomassie Brilliant Blue R250, then de‐stained, followed by imaging.

### 2.10 Confocal microscopy

Immunofluorescence staining was performed with anti‐ETS‐1, anti‐HIF‐1α and anti‐E‐cadherin sera (1 : 100) followed by Alexa‐Fluor 488‐conjugated secondary antibody (1 : 200), as described previously (Basu *et al*. (2015). Phalloidin‐tetramethylrhodamine B isothiocyanate (TRITC) (Sigma) staining was performed as described by Faulstich *et al*. (1983). Images were taken using an Andor Spinning Disk Confocal Microscope.

### 2.11 Flow‐cytometric intracellular antigen staining

Cells treated with LPA for 24 h (with and without receptor inhibitor) were used in fluorescence‐activated cell sorting analysis (Jacobberger *et al*., 1986). Briefly, cells were fixed with 0.5% paraformaldehyde for 15 min on ice, permeablized by adding ice‐cold 70% ethanol drop‐wise with constant mixing, and kept at 4°C for 30 min. Cells were incubated with anti‐ETS‐1 serum (1 : 200) for 2 h at room temperature, then washed, followed by incubation with anti‐FITC (1 : 600) for 1 h at room temperature and analyzed using a LSR Fortessa cell analyser (BD Biosciences).

### 2.12 Chromatin immunoprecipitation

Chromatin immunoprecipitation (ChIP) with cells overexpressed/treated for 24 h was performed using a ChIP kit (Upstate, Temecula, CA, USA) following the protocol described by Basu *et al*. (2015). Equal amounts of the immunoprecipitate and input were used for ChIP–PCR with the following the conditions: 95 °C for 30 s, annealing at specific temperature for each set of primers for 30 s and 72 °C for 20 s, for 30 cycles. The sequences and other information regarding the primers are given in Table 2.

**Table 2:** Sequence of the oligonucleotide primers used in ChIP–PCR

Gene product	Forward primer (5′–3′)	Reverse primer (5′–3′)	Tm (°C)
*H_ets‐1*	GCACTGGTTTACTGCTTGCT	GGCAGGGAAAGCAATGGAAA	59
*H_mmp2*	CATTGTCAATGTTCCCTAAAACATTC	CTCCCTCTCTCAGGAAAGACAGTTG	55
*H_mmp9*	TGCGGACTTACAACCTACAGTG	TCTTTGACTCAGCTTCCTCTCC	55

### 2.13 3‐(4,5‐dimethylthiazol‐2‐yl)‐2,5‐diphenyltetrazolium bromide (MTT) assay for cell proliferation

Treated cells were incubated with 10 μL MTT reagent (Invitrogen) and kept in the dark for 2–3 h. The reaction was stopped with dimethyl sulfoxide and the absorbance was measured at 570 nm. Percent cell viability was calculated as (treated/control)* × *100.

### 2.14 RNA sequencing and analysis

Total RNA was isolated as mentioned previously. Global transcriptomic sequencing was performed at Genotypic Technology Pvt. Ltd (Bangalore, India). The RNA‐sequencing raw data files were deposited in the NCBI Gene Expression Omnibus (GEO) and are accessible under GEO series accession number GSE87087. Using Fastq files, all the sequencing reads were mapped to the human genome using tophat (v. 2.0.11). cufflinks (v. 2.2.1) was used to assemble the mapped reads from RNA‐Seq against ENSEMBL gene structure annotation, and the expression level for each transcript was estimated. A cufflinks‐based probabilistic model of paired‐end sequencing was used to evaluate the abundance of each isoform. To analyze gene expression, estimated expression levels were converted from FPKM units (number of reads × 10^9^/transcript length/library) to pseudocounts (number of reads originating from each transcript isoform). Differentially expressed transcripts were identified using the CuffDiff protocol. A fold change of two and above with a *p*‐value of ≤ 0.05 was used as a cut‐off to identify differentially expressed genes. Unsupervised hierarchical clustering of differentially expressed genes was done using the Pearson uncentered correlation with average linkage rule using cluster v. 3.0 software and visualized using Java treeview software. Functional annotation (Gene Ontology and Pathways) clustering of the differentially expressed transcripts was carried out using the DAVID web server with ‘Medium’ default settings for classification stringency. Biological network analysis of differentially expressed transcripts and the gene ontology (GO)/pathway harboring them were performed using bridge island software (Bionivid Technology Pvt Ltd, Bangalore, India). The resultant file comprising nodes and edges was imported into cytoscape v. 2.8 to visualize the regulatory network comprising differentially expressed transcripts and the connecting GO/pathway.

### 2.15 Animal models

Female BALB/c mice (*n* = 10; 4–6 weeks old) were injected subcutaneously in the right flank with 5 × 10^6^ ID8 cells in 100 μL PBS along with an equal volume of Matrigel. Subcutaneous LPA injections (0.4 μmol per 100 μL PBS) were given in the area containing the cells in one set of mice (*n* = 4), whereas another set received only 100 μL PBS (*n* = 4) every other day. A control set was treated only with 100 μL PBS. Tumors were harvested after 20 days and tumor volume was measured as (length × width^2^) × 0.5. Relative tumor volume was plotted in a bar diagram. One portion of the tumors was fixed in 10% formalin for immunohistochemistry and another was kept in PBS for western analysis. All animal experimental procedures were approved by the animal ethics committee of CSIR‐Indian Institute of Chemical Biology for the Purpose of Control and Supervision of Experiments on Animals (CPCSEA), Ministry of Culture, Government of India (Sanction number: 147/1999/CPCSEA and IICB/AEC‐APP/March‐Meeting/2014). The experiments were executed in accordance with proper guidelines and regulations of this Institution and relevant committees.

### 2.16 Fluorescence immunohistochemistry

The primary tumor was sectioned, fixed, processed and stained as mentioned previously (Basu *et al*., 2015). Tissue sections were overlaid with blocking solution (5% bovine serum albumin‐1XTBS‐T) for 30 min and incubated overnight with anti‐ETS‐1 and anti‐HIF‐1α (1 : 100) sera. Slides were then washed and incubated for 2 h in the dark with secondary anti‐Alexa Fluor‐488 (1 : 500) followed by 4′,6‐diamidino‐2‐phenylindole (DAPI) staining. Images were captured using an Andor Spinning Disk Confocal Microscope.

### 2.17 Statistical analysis

All data are expressed as mean ± SEM, and statistical analyses were performed using the Student's *t*‐test; a *p*‐value of < 0.05 was considered significant, unless otherwise stated. The experiments were repeated at least three times unless otherwise specified.

## 3 Results

### 3.1 LPA promotes bioenergetic modifications in ovarian cancer cells

**Figure 1:**
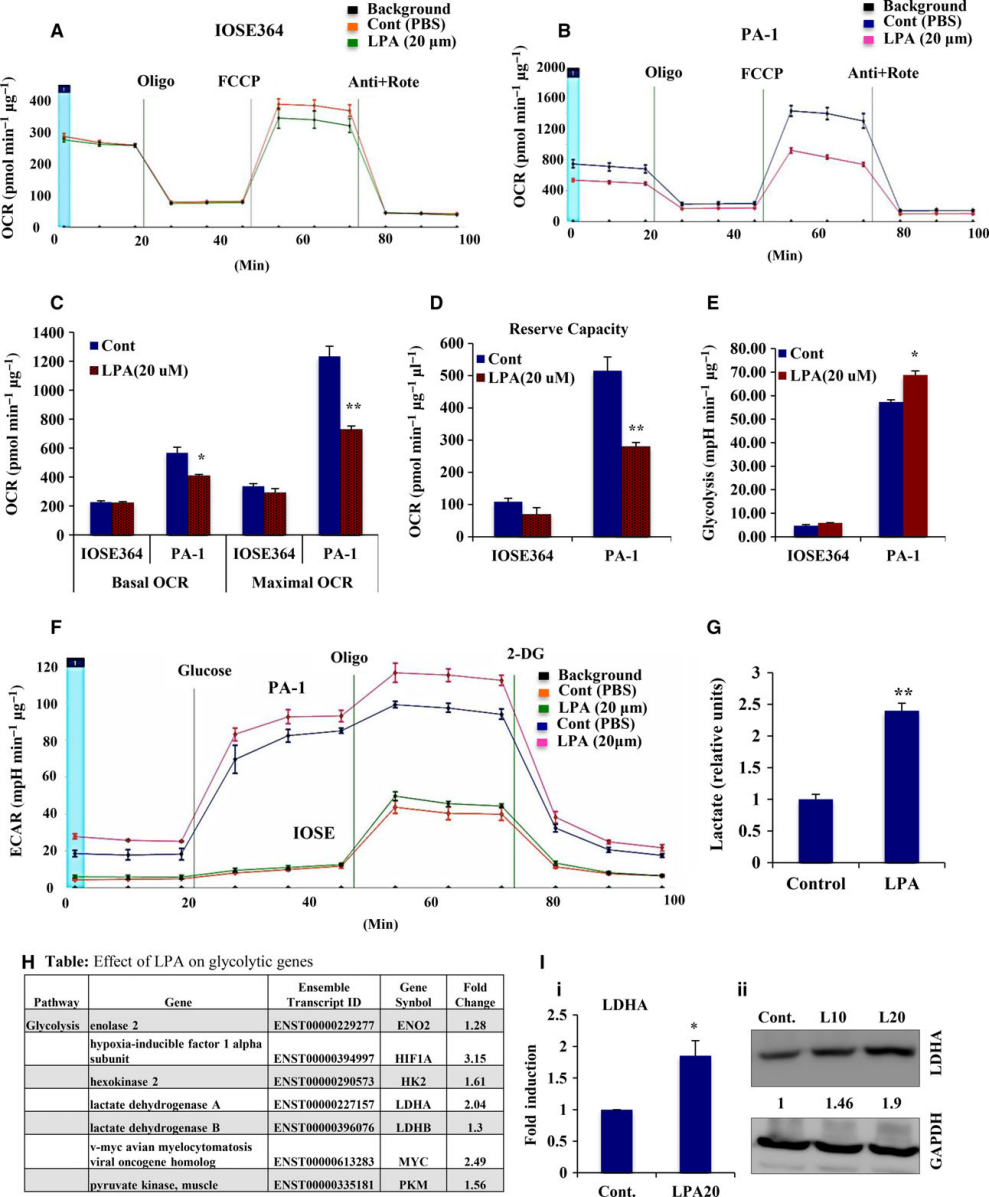
LPA is associated with metabolic modulation of ovarian cancer (OC) cells. (A,B) OCRs (pmol·min^−1^·μg^−1^) were determined in both non‐cancer IOSE‐364 and PA‐1 OC cell lines using Seahorse XF‐24 flux analyzer upon exposure to LPA for 24 h. Oligomycin, FCCP, and antimycin A + rotenone were sequentially administered as indicated. (C) Basal OCR was calculated from the measurement prior to oligomycin addition minus non‐mitochondrial respiration, whereas maximal OCR was calculated from FCCP‐induced levels minus non‐mitochondrial respiration. Results are represented as mean ± SEM (*p < 0.05, **p < 0.01). (D) Reserve capacity was obtained as maximal minus basal OCR (**p < 0.01). (E) Glycolytic rate (mpH·min^−1^·μg^−1^) was determined and plotted from the ECAR measurements (F) in both IOSE‐364 and PA‐1 OC cell lines in response to LPA for 24 h. Glucose, oligomycin and 2‐deoxyglucose were injected sequentially to the cells in glucose‐free medium. Glycolytic rate was calculated by deducting the measurement after glucose injection (prior to oligomycin injection) from the measurement prior to glucose injection. Results are provided as mean ± SEM (*p < 0.05). (G) Quantitative ELISA was used to measure cellular lactate production in the conditioned medium from the treated cells. The result was given as fold change with respect to non‐treated cells in bar graph (**p < 0.01). (H) The effect of LPA on some key glycolytic genes was analyzed from the RNA‐Seq data. For the given gene sets of glycolysis, the analysis was achieved with the transcripts having greatest difference in control values, mentioned as form of fold change. This was analyzed by ranking genes by normalized expression or raw counts, and those taken with the greatest difference in observed values. (I) LDHA expression levels analyzed in LPA‐treated cells compared with non‐treated controls by quantitative PCR (i, *p < 0.05) and western detection (ii). Fold change for the protein expression was calculated using image j software (normalized against GAPDH) and is provided beneath the panel.

Because previous reports have established LPA as an essential mediator of cancer progression, we aimed to study its effect on metabolic modulation in ovarian cancer cells. The oncogenic potential of cancer cells depends on their adaptation to aerobic glycolysis for more energy, therefore we investigated the effect of LPA on the bioenergetic status of the non‐transformed IOSE‐364 and the PA‐1 ovarian cancer cells using a Seahorse XF‐flux analyzer (Fig. 1A,B). No significant effect on basal OCR was found for non‐cancer ovarian epithelial cells, whereas an evident reduction in OCR was observed in ovarian cancer cells in the presence of LPA (Fig. 1C). Furthermore, significant attenuation in maximal OCR was found in PA‐1 cells compared with non‐transformed cells, suggesting reduced mitochondrial oxidative phosphorylation in ovarian cancer cells (Fig. 1C). Moreover, a significant decrease in the calculated OCR reserve capacity was obtained after LPA treatment, suggesting a cellular predisposition from mitochondrial activity towards glycolysis in ovarian cancer cells (**p < 0.01, Fig. 1D). To further validate the glycolytic predisposition of these treated cells, we analyzed the ECAR in both cell types (Fig. 1E,F). A significant increase in the glycolysis rate was observed upon LPA treatment in PA‐1 compared with IOSE cells. In addition, enrichment at the level of lactate production was seen in LPA‐treated ovarian cancer cells (Fig. 1G).

This effect of LPA on metabolic adaptation prompted us to investigate the underlying gene regulation. We performed high‐coverage deep sequencing of the transcriptome upon LPA (20 μm) induction for 24 h in highly aggressive PA‐1 ovarian cancer cells (GEO series accession number GSE87087). Focusing on glycolytic gene signatures in response to LPA, we found significant upregulation of several key glycolytic genes (Figs 1H and S1A). Enhancement of the expression of LDHA, HK2 and PKM2 genes was further noted (Figs 1I and S1B). This activated glycolytic dependency of LPA‐induced ovarian cancer cells improves their energetic status, which might be a plausible explanation for the increased invasive potential of ovarian cancer cells.

### 3.2 Long exposure to LPA defines the transcriptome to specify invasive fate

**Figure 2:**
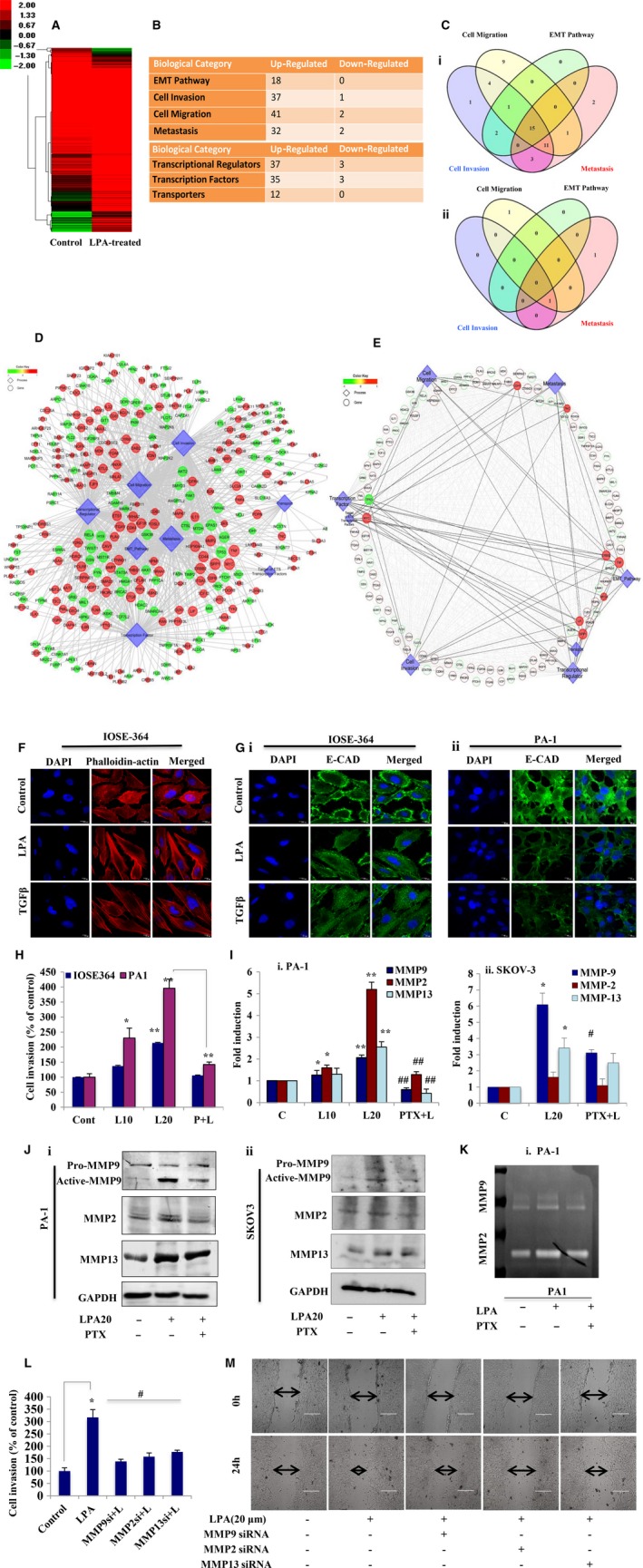
Gene‐regulatory networking unfolds modification of the LPA‐treated ovarian cancer (OC) cell transcriptome towards increased invasion. (A) Heat map showing standardized expression (*Z*‐scores) of genes differentially expressed upon long induction of LPA (20 μm) for 24 h in PA‐1 cells. (B) GSEA analysis revealing biological processes that promote cancer and gene families involved in signal transduction, transcriptional and translational regulation (*p* ≤ 0.05) on LPA treatment. (C) Venn diagram showing distribution of (i) upregulated and (ii) downregulated genes involved in multiple biological processes that promote tumor progression. (D) Global gene regulatory network encompassing differentially expressed genes and the key pathways involved. The genes are colored based on their fold change and the size is based on their connectivity score. (E) Core regulatory network encompassing key differentially expressed genes and the selective pathways involved, based on criteria as above. (F) IOSE cells were treated with LPA to show actin rearrangement resembling the mesenchymal phenotype through confocal microscopy after TRITC–phalloidin staining. Scale bar = 10 μm. (G) Confocal imaging for E‐cadherin expression was performed in (i) IOSE and (ii) PA‐1 cells after LPA treatment. In all cases, DAPI was used to stain the nucleus and merged images are shown. Scale bar = 10 μm. TGF‐β was used as a positive inducer of EMT in all cases. (H) Transwell invasion assay performed in PA‐1 and IOSE cells after treatment with LPA in the absence and presence of PTX for 22 h. Cells at three independent fields for each well were counted and are plotted with error bars (*p < 0.05, **p < 0.01). (I) MMP‐2, MMP‐9 and MMP‐13 mRNA expression was analyzed by quantitative PCR after treatment with LPA in the absence and presence of PTX (*p < 0.05, **p < 0.01 vs control; #*p* < 0.05, ##*p* < 0.01 vs LPA treatment) in (i) PA‐1 and (ii) SKOV‐3 cells. (J) Western blot analysis for the respective MMPs was performed with treatments similar to those mentioned for (i) PA‐1 and (ii) SKOV‐3 cells. (K) Gelatin zymography for MMP‐2/MMP‐9 activity assay in PA‐1 cells. (L) Matrigel invasion assay performed after cells were transfected with the indicated siRNAs followed by LPA induction for 22 h. Percent cell invaded was counted at three independent fields for each well (*p < 0.05 vs control, #*p* < 0.05 vs LPA treatment). (M) Wound healing assay performed with similar treatment to that described. Images at 0 and 24 h. Scale bar = 200 μm.

RNA‐Seq analysis revealed an average of 15 million high‐quality sequence reads in both control and treated samples that mapped to the human reference genome GrCH38, resulting in the identification of 39 255 transcripts expressed in either the control or treated sample (GSE87087). Heat map clustering of differentially expressed transcripts suggests a high degree of induction in response to LPA (Fig. 2A). Further gene set enrichment analysis (GSEA) revealed significant induction of biological processes like EMT, invasion and metastasis, and the associated gene families (*p* ≤ 0.05, Fig. 2B). Additionally, the Venn diagram suggests significant upregulation of invasion‐ and metastasis‐related genes (Fig. 2Ci,ii). This influence of LPA on the global aggressive signature led us to investigate the essential gene regulatory events. Accordingly, the differentially expressed transcripts, key pathways and gene ontologies were studied to identify key nodes and edges that encompass the regulatory circuit underlying the LPA‐induced changes. The resultant network clustered genes based on their connectivity score to identify key nodes that are enriched (Fig. 2D). Further detailed analysis of the core network revealed that key driver genes like TP53 are downregulated, whereas MYC, ERBB2, LIF, SPP1 and MEF2A are upregulated controlling pathways like EMT, invasion and metastasis (Fig. 2E). These results suggest a potential invasive signature through LPA‐mediated transcriptional modulation.

To address the invasive potential of this oncolipid, we determined its physiological effect in non‐transformed cells. Actin rearrangement towards the mesenchymal phenotype was observed by TRITC–phalloidin staining in LPA‐treated IOSE cells (Fig. 2F). A significant increase in the expression of the mesenchymal marker genes N‐cadherin (*CDH2*) and fibronectin (*FN*), with subsequent reduction in the epithelial marker genes E‐cadherin (*CDH1*) and claudin‐7 (*CLDN7*), was observed in non‐transformed cells (Fig. S2A,B). The E‐cadherin level was also found to decrease, as shown by immunostaining in LPA‐treated IOSE and PA‐1 cells compared with controls (Fig. 2Gi,ii). The Matrigel invasion assay showed an approximately twofold increase in the invasion of PA‐1 compared with non‐cancer cells upon treatment with 20 μm LPA; the invasiveness was reduced by the LPA receptor inhibitor PTX (Figs 2H and S2C). Furthermore, long exposure to LPA leads to an increase in wound closure to ~ 70% at 24 h compared with controls in PA‐1 cells (Fig. S2D). A comparable pattern of enhanced invasion (Fig. S2E) was also observed in OAW‐42 ovarian cancer cells under similar conditions.

We investigated the status of important effector genes involved in tumor progression. Heat map analysis of effector genes showed that distinct members like MMP‐2 and MMP‐7 were differentially upregulated by LPA in PA‐1 cells (Fig. S2F). However, in subsequent follow‐up experiments, we found significant upregulation in the expression of MMP‐9 and MMP‐13 in PA‐1, SKOV‐3 (Fig. 2Ii,ii and Ji,ii) and OAW‐42 (Fig. S2G,H) cells upon LPA treatment, which is reduced in the presence of its receptor inhibitor. Conversely, MMP‐2 was found to be significantly increased (approximately fivefold) by LPA only in PA‐1 cells, thereby suggesting a cell‐type specific pattern of expression and function (Fig. 2Ii and Ji). Increased activity of MMP‐9 was observed in both the ovarian cancer cells, whereas MMP‐2 activity was increased only in PA‐1 cells (Figs 2K and S2I), as shown by gelatin zymography. To determine the functional significance of these proteinases, we transfected cells with MMP‐2, MMP‐9 and MMP‐13‐specific siRNAs, which showed a significant decrease in LPA‐induced invasion (Figs 2L and S2J) and migration (Fig. 2M) in PA‐1 cells. Taken together these results strongly suggest the oncogenic potential of LPA towards increased ovarian cancer invasiveness.

### 3.3 Activation of the AKT pathway is a critical determinant of LPA‐induced invasiveness

RNA‐Seq analysis revealed multiple signaling pathways to be dysregulated, prompting us to identify the driver signaling axes activated upon LPA treatment. Among others, the PtdIns3K–AKT cascade is significantly activated in response to LPA (Fig. S2A), which led us to further investigate the role of this pathway.

**Figure 3:**
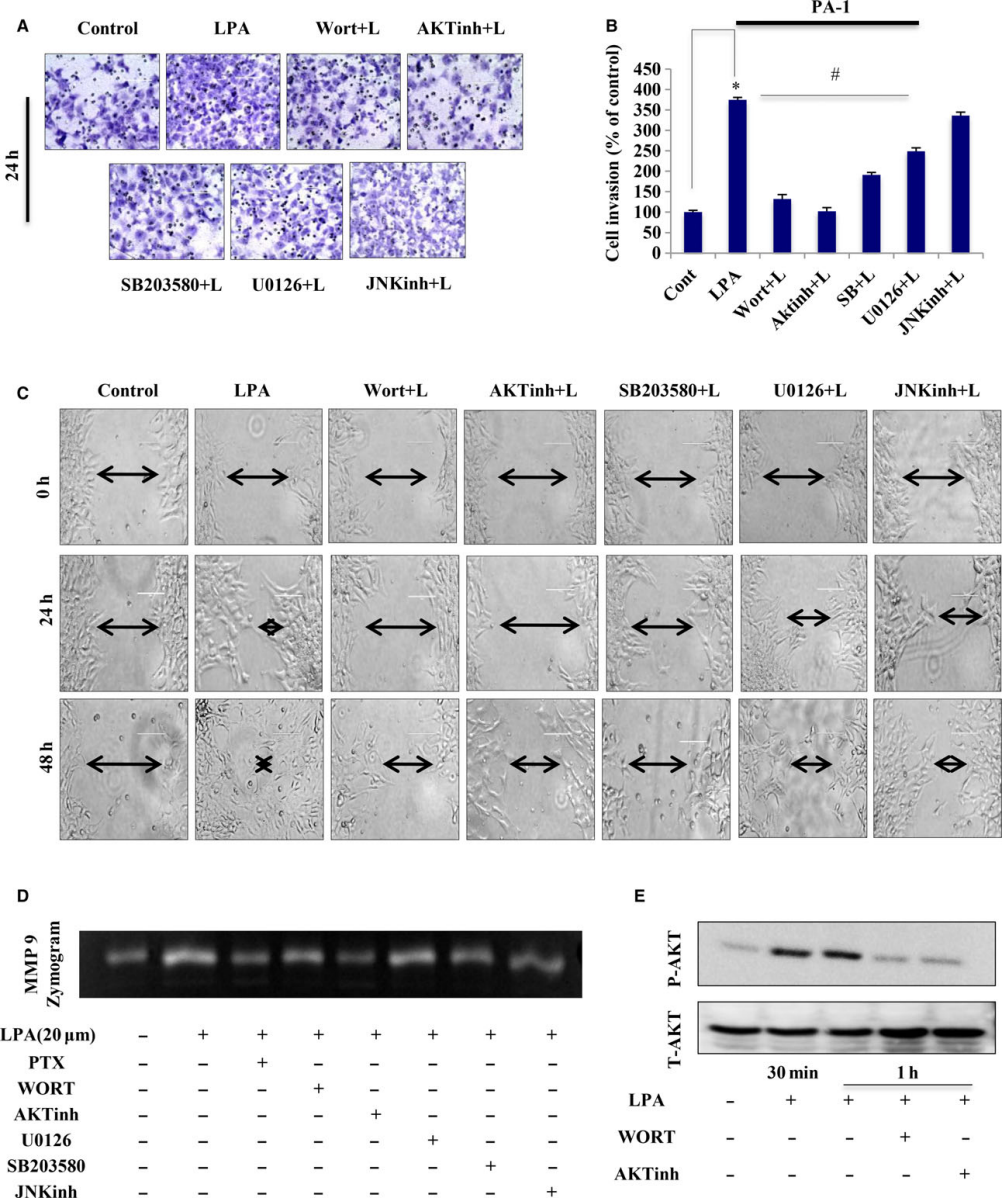
AKT pathway activation is critical for LPA‐induced ovarian cancer (OC) invasion. (A) Matrigel invasion assay performed with PA‐1 cells pretreated with the indicated pathway inhibitors for 1 h followed by LPA for 22 h. Scale bar = 100 μm. (B) Percent cell invasion plotted with cells counted in three independent fields for each well (*p < 0.05 vs control, #*p* < 0.05 vs LPA treatment). (C) Scratch wound healing assay performed with the mentioned treatments for 24 and 48 h. 0 h images are provided. Scale bar = 100 μm. Arrows indicate the width of the wound and the assay was repeated three times. (D) MMP‐9 activity was analyzed by gelatin zymography with the treatments as mentioned. (E) AKT pathway activation was performed with the indicated treatments by analyzing p‐AKT and total AKT levels in a western blot.

To explore this pathway, we analyzed the effect of selective inhibitors of key signaling pathways that are mostly activated during cancer progression. Specific inhibition of the PtdIns3K and AKT pathway leads to significant attenuation of the LPA‐induced invasion in PA‐1 (approximately twofold, Fig. 3A,B) and OAW‐42 (~ 1.5‐fold, Fig. S2B) cells compared with p38MAPK, MEK and JNK inhibition. A similar reduction in migration was observed upon inhibition of the PtdIns3K–AKT pathway (Fig. 3C). To gain insight, MMP‐9 activity was assessed, and showed maximum reduction in the presence of AKT/PtdIns3K‐specific inhibitors compared with others (Fig. 3D). Activation of AKT signaling was further verified by analysis of p‐AKT levels following LPA administration, which are reduced in the presence of PtdIns3K and AKT inhibitor (Fig. 3E). Overall, the data reveal the importance of PtdIns3K–AKT signaling activation as a critical determinant of the LPA‐induced invasive fate of ovarian cancer cells.

### 3.4 LPA‐signaling induces a repertoire of oncogenic transcription factors

**Figure 4:**
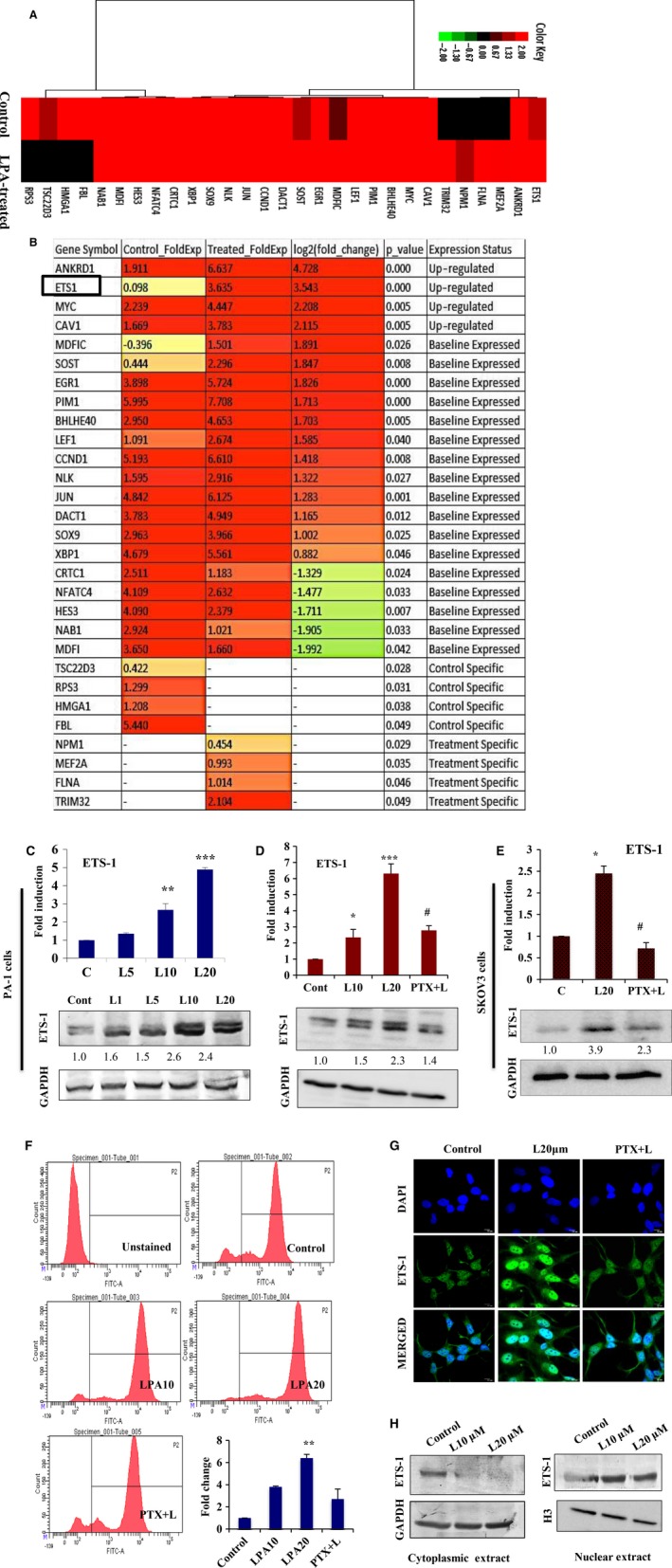
LPA induces ETS‐1 expression. (A) Heat map presenting clustering of all differentially expressed transcriptional regulators on LPA treatment. (B) Statistical ranking of the induced transcription factors were shown with fold change and *p*‐value. (C) Quantitative PCR (*p < 0.05, ***p < 0.001) and western blot were examined with 1, 5, 10 and 20 μm LPA on ETS‐1 expression. (D) Expression of ETS‐1 was analyzed by both quantitative PCR (*p < 0.05, ***p < 0.001 vs control; #*p* < 0.05 vs LPA treatment) and immunoblot upon treatment of PA‐1 cells with LPA in the absence and presence of PTX. (E) ETS‐1 expression was further analyzed in SKOV‐3 cells under similar treatments. In all cases, fold change for the protein expression was calculated using image j software (normalized against GAPDH) and is provided beneath the panel. (F) Quantitative expression of ETS‐1 was analyzed in PA‐1 cells by flow cytometry with the indicated treatments. Fold change with respect to control was plotted (**p < 0.01). (G) LPA‐induced nuclear localization of ETS‐1 in the absence and presence of PTX using confocal microscopy. DAPI is used to stain the nucleus and merged images are provided. Scale bar = 10 μm. (H) Nuclear and cytoplasmic distribution of ETS‐1 upon dose‐dependent exposure to LPA (0, 10 and 20 μm) for 24 h. GAPDH and histone H3 were used as the loading control for the cytoplasmic and nuclear fractions, respectively.

We next attempted to elucidate the central regulatory factors governing oncolipid‐mediated functions. Clustering of differentially expressed transcription factors showed that ETS‐1, along with a battery of known key regulators like MYC, LEF1 and SOST, is significantly upregulated on treatment (Fig. 4A). Statistical ranking of the induced transcription factors revealed that ETS‐1 was significantly enriched (approximately threefold, *p* < 0.001, Fig. 4B). We further analysed 12 subfamilies of the ETS group of transcription factors, and found that ETS‐1 is more significantly upregulated than other transcription factors in LPA‐induced ovarian cancer cells (Fig. S4A).

To validate these results, we found a dose‐dependent significant enhancement in the expression of ETS‐1 with increased LPA concentration (maximum approximately fivefold at 20 μm, Fig. 4C). Treatment in the presence of the LPA receptor inhibitor PTX showed a reduction in the levels of LPA‐induced ETS‐1 in PA‐1, SKOV‐3 (Fig. 4D,E) and OAW‐42 cells (Fig. S4B). Insignificant changes in the expression of ETS‐2 belonging to the ETS subfamily were found under similar conditions (Fig. S4C). Further quantitation of LPA‐induced ETS‐1 expression by flow cytometry also showed significant upregulation (Fig. 4F). In addition, immunostaining followed by confocal microscopy (Fig. 4G) and nuclear/cytoplasmic protein fate analysis (Fig. 4H) revealed enriched nuclear translocation of ETS‐1 in response to LPA. We also found upregulation of ETS‐1 expression in non‐malignant IOSE cells upon chronic exposure to LPA for 24 h (Fig. S4D). Collectively, these data strongly suggest that ETS‐1 is a critical transcription factor to be induced in response to LPA‐signaling in ovarian cancer cells.

### 3.5 Depletion of ETS‐1 attenuates LPA‐induced bioenergetic reprogramming and prevents invasion

**Figure 5:**
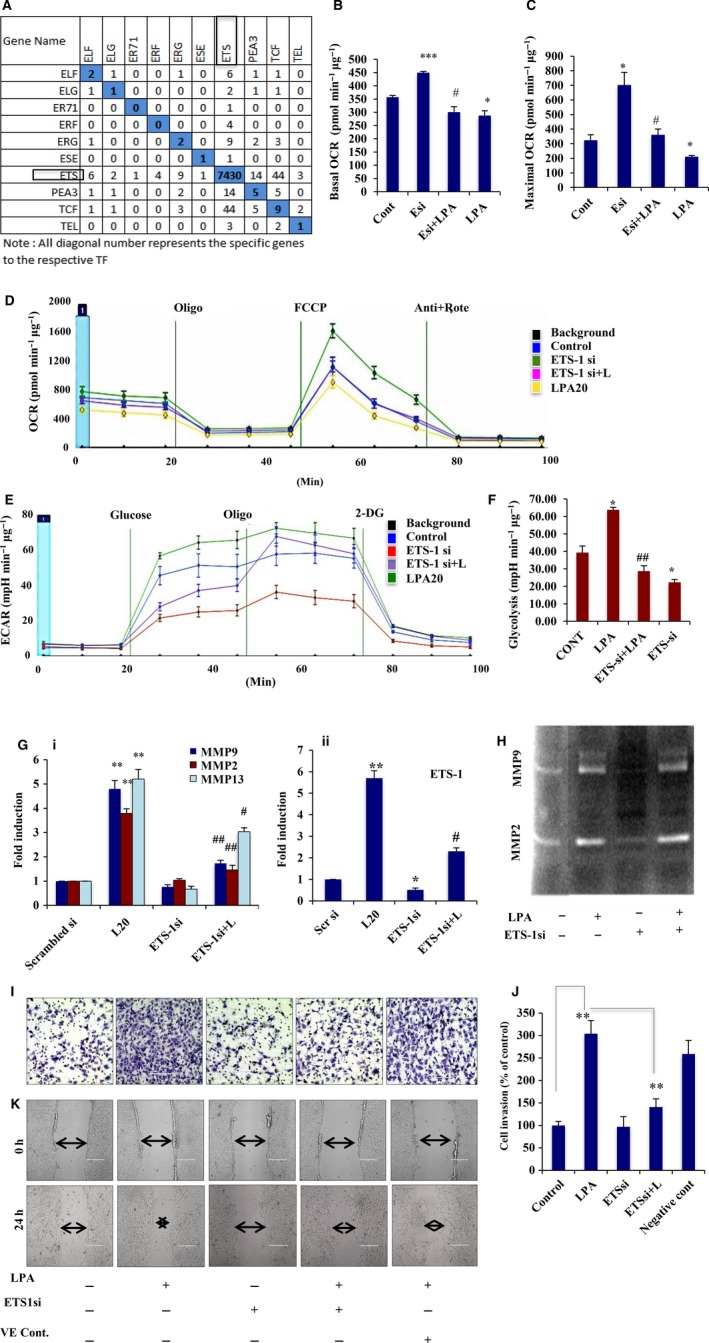
ETS‐1 knockdown abrogates LPA‐induced metabolic reorientation. (A) Gene matrix representing the common target transcripts expressed under the ETS subfamily members. All diagonal numbers represent specific genes expressed by the respective transcription factors. Representative (B) basal and (C) maximal OCR (pmol·min^−1^·μg^−1^) are calculated and plotted from the OCR analysis (D) in ETS‐1 knockdown ovarian cancer cells in the presence and absence of LPA using a Seahorse XF‐24 extracellular flux analyzer. Both graphs were plotted after deduction of the non‐mitochondrial respiration; error bars indicate SEM (*p < 0.05, ***p < 0.001 vs control; #p < 0.05 vs ETS‐1 knockdown). (E) ECAR analysis was obtained from similar treatment of the cells in glucose‐free medium and (F) the glycolytic rate (mpH·min^−1^·μg^−1^) was plotted. Glycolytic rate was analyzed by subtracting values obtained after glucose addition (prior to oligomycin) from those obtained prior to glucose injection; error bars indicate SEM (*p < 0.05 vs control; ##p < 0.01 vs LPA treatment). (G) PA‐1 cells were transfected with ETS‐1 siRNA and then treated with/without LPA followed by quantitative PCR analysis against MMP‐2, MMP‐9 and MMP‐13 expressions (**p < 0.01 vs control; #p < 0.05, ##p < 0.01 vs LPA treatment). Downregulation of ETS‐1 was also confirmed by quantitative PCR analysis in these cells. (H) MMP‐2/MMP‐9 activity was shown by gelatin zymography in the cells treated as mentioned. (I,J) Effect of ETS‐1 knockdown on invasion of PA‐1 cells in the presence/absence of LPA. Scrambled siRNA transfected cells were treated as a negative control. The invasion experiments were performed three times, and the mean ± SEM (error bars) plotted (**p < 0.01). (K) Similar transfection was performed to show the migration phenomenon in PA‐1 cells by wound closure assay. 0 and 24 h images are given. Scale bar = 200 μm.

Driven by observations that suggest induction of ETS‐1 is the key regulatory event induced upon treatment with LPA, we analyzed the expression status of its target genes. Matrix representation revealed enrichment of 7430 target genes regulated by ETS‐1 (Fig. 5A).

To address the contribution of ETS‐1 to the shift in the bioenergetics observed in ovarian cancer cells upon LPA induction, we analyzed the effect of ETS‐1 knockdown in these cells. Basal and maximal OCR were found to be significantly higher in the ETS‐1 knockdown cells compared with non‐targeted control. However, treatment with LPA in these knockdown cells reversed the shift in OCR (Fig. 5B–D). In addition to this, ECAR analysis revealed a decrease in the glycolytic phenomenon in ETS‐1 knockdown cells, which is compromised in presence of LPA (Fig. 5E,F). In addition, ETS‐1 knockdown was found to reduce the expression of LDHA and pyruvate kinase M2 in LPA‐induced PA‐1 cells (Fig. S4E). Thus, this oncolipid leads to a metabolic shift towards a more glycolytic phenotype, promoting the invasive potential of the transformed cells, which is reversed upon silencing of ETS‐1.

The functional significance of ETS‐1 was further investigated by assessing the effect of its knockdown on the LPA‐induced invasion and migration of ovarian cancer cells. Because LPA was found to significantly upregulate MMP‐2, MMP‐9 and MMP‐13, we determined their fate upon ETS‐1 silencing. We observed a significant reduction in the LPA‐mediated expression and activity of these MMPs in ETS‐1 knockdown cells (Fig. 5G,H). Furthermore, ChIP with anti‐ETS‐1 IgG followed by PCR showed amplifications of the *mmp‐2/‐9* promoter, indicating enriched binding of ETS‐1 with the respective promoter upon exposure to LPA (Fig. S4F). Significant attenuation was also observed in invasion (~ 1.5‐fold, Fig. 5I,J) and migration (Fig. 5K) of the ETS‐1 knockdown cells compared with LPA treatment. Together, these data certify the involvement of ETS‐1 to increase tumorigenesis in ovarian cancer cells.

### 3.6 LPAR2‐specific AKT activation is crucial for LPA‐induced ETS‐1 expression

**Figure 6:**
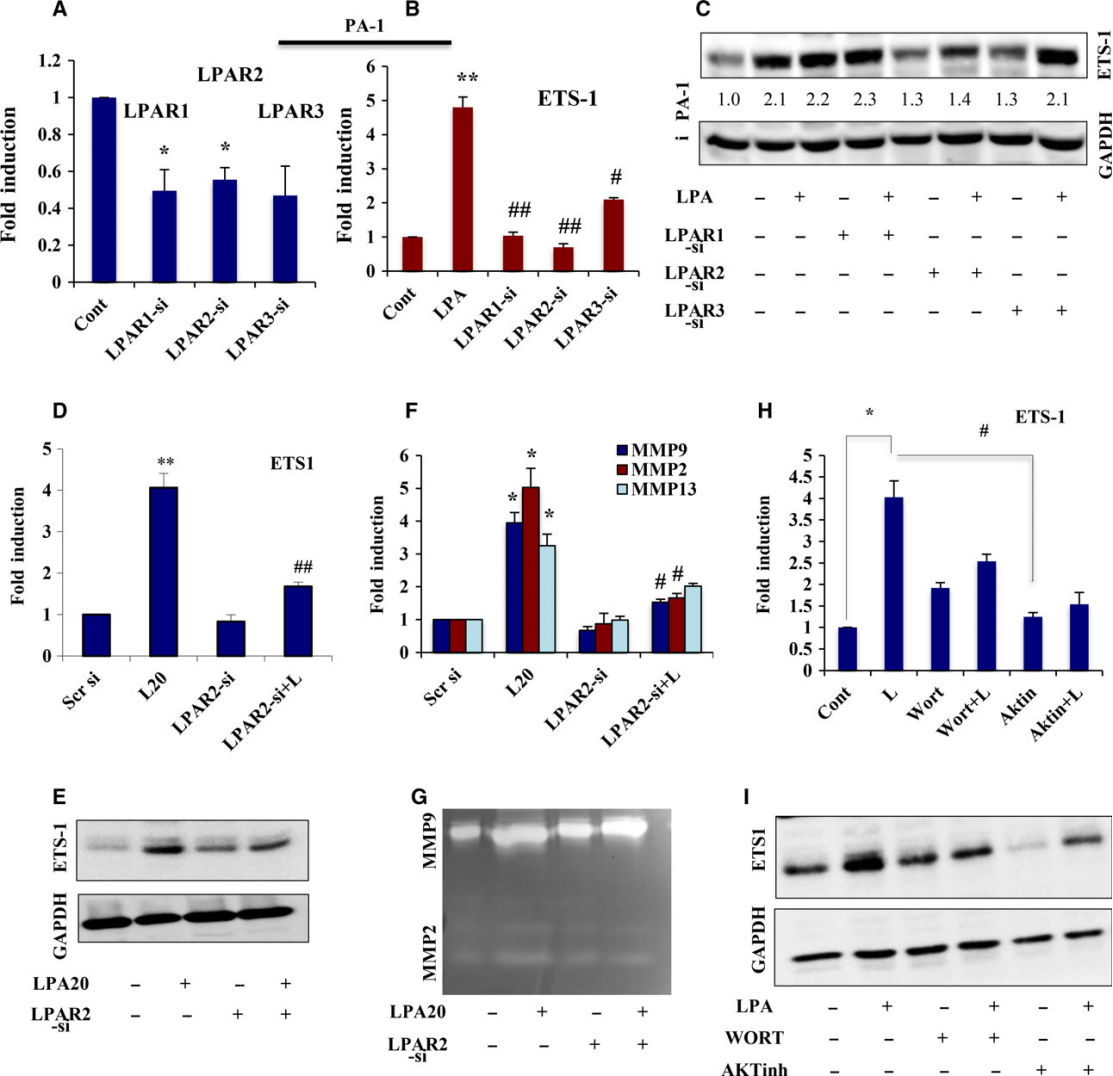
LPAR2‐mediated induction of AKT‐signaling is involved in ETS‐1 expression. (A) Quantitative PCR was performed to show the ability of each of the three LPA receptor‐specific siRNAs (LPAR1/2/3) to significantly knockdown their own expression in PA‐1 cells. (B) ETS‐1 expression level was analyzed in these knockdown cells as indicated (**p < 0.01 vs control; #*p* < 0.05 and ##*p* < 0.01 vs LPA treatment). (C) PA‐1 cells were transfected with the indicated LPA receptor‐specific siRNAs, serum starved and stimulated with 20 μm LPA for 24 h, followed by western analysis of ETS‐1. GAPDH was used as a loading control. The densities of the respective bands were calculated using image j software (normalized against GAPDH) and are represented as ‘fold change’ beneath the panel. (D) LPAR2‐siRNA transfected PA‐1 cells treated with/without LPA followed by quantitative PCR analysis. Results show the mean ± SEM (**p < 0.01 vs control; ##*p* < 0.01 vs LPA treatment). (E) Western blot analysis of ETS‐1 expression was done following similar treatment. (F) Quantitative PCR analysis of MMPs was performed in the same transfected cells following the mentioned treatments (*p < 0.05 vs control, #*p* < 0.05 vs LPA treatment). (G) MMP‐2/MMP‐9 activity was assessed by gelatin zymography in the conditioned media of the transfected cells. (H) Quantitative PCR (*p < 0.05 vs control, #*p* < 0.05 vs LPA treatment) and (I) immunoblot analysis of ETS‐1 expression was performed in PA‐1 cells pretreated with PtdIns3K/AKT inhibitors for 1 h followed by LPA induction.

Given that LPAR1/2/3 expression is linked to invasion and metastasis in different cancer types, we investigated the specific receptor subtype responsible for ETS‐1 regulation in ovarian cancer cells. Expression of the three receptors in the two cell types was first validated using PCR analysis (Fig. S5A). siRNA‐mediated knockdown of LPAR2, but not LPAR1/3 significantly inhibited LPA‐induced ETS‐1 expression in PA‐1 cells (Fig. 6A–C). Knockdown of LPAR2 in OAW‐42 and LPAR2/3 in SKOV‐3 cells resulted in abrogation of the LPA‐mediated ETS‐1 upregulation (Fig. S5B,C). To further confirm this, we knocked down LPAR2 and found significant attenuation in the expression of both LPA‐induced ETS‐1 (Fig. 6D,E) and subsequent downstream MMPs (Fig. 6F,G) in PA‐1 cells. Overall, these data suggest LPAR2‐specific regulation of invasion in ovarian cancer cells through ETS‐1. Furthermore, involvement of the AKT pathway was verified by treatment with AKT inhibitor, which showed significant reduction in the expression of LPA‐induced ETS‐1 (Fig. 6H,I). Taken together, these results confirm the importance of the aberrant activation of AKT signaling to oncolipid‐mediated aggressiveness through the Gi‐LPAR2 axis.

### 3.7 LPA stimulation creates pseudo‐hypoxia in ovarian cancer cells

Having shown that ETS‐1 is a key oncogenic factor in metabolic adaptations towards increased aggressive potential in ovarian cancer, we further wanted to identify its regulatory partners. Several reports have indicated the coexpression of HIF‐1α and ETS‐1 in cancer. Treatment with CoCl_2_ mimics hypoxia through stabilization of HIF‐1α expression (Fig. S6A). We found upregulation of ETS‐1 (Fig. S6B,C) by CoCl_2_ treatment, which is reduced upon silencing of HIF‐1α (Fig. S6D,E), suggesting that there might be some existing regulation between ETS‐1 and HIF‐1α in cancer.

**Figure 7:**
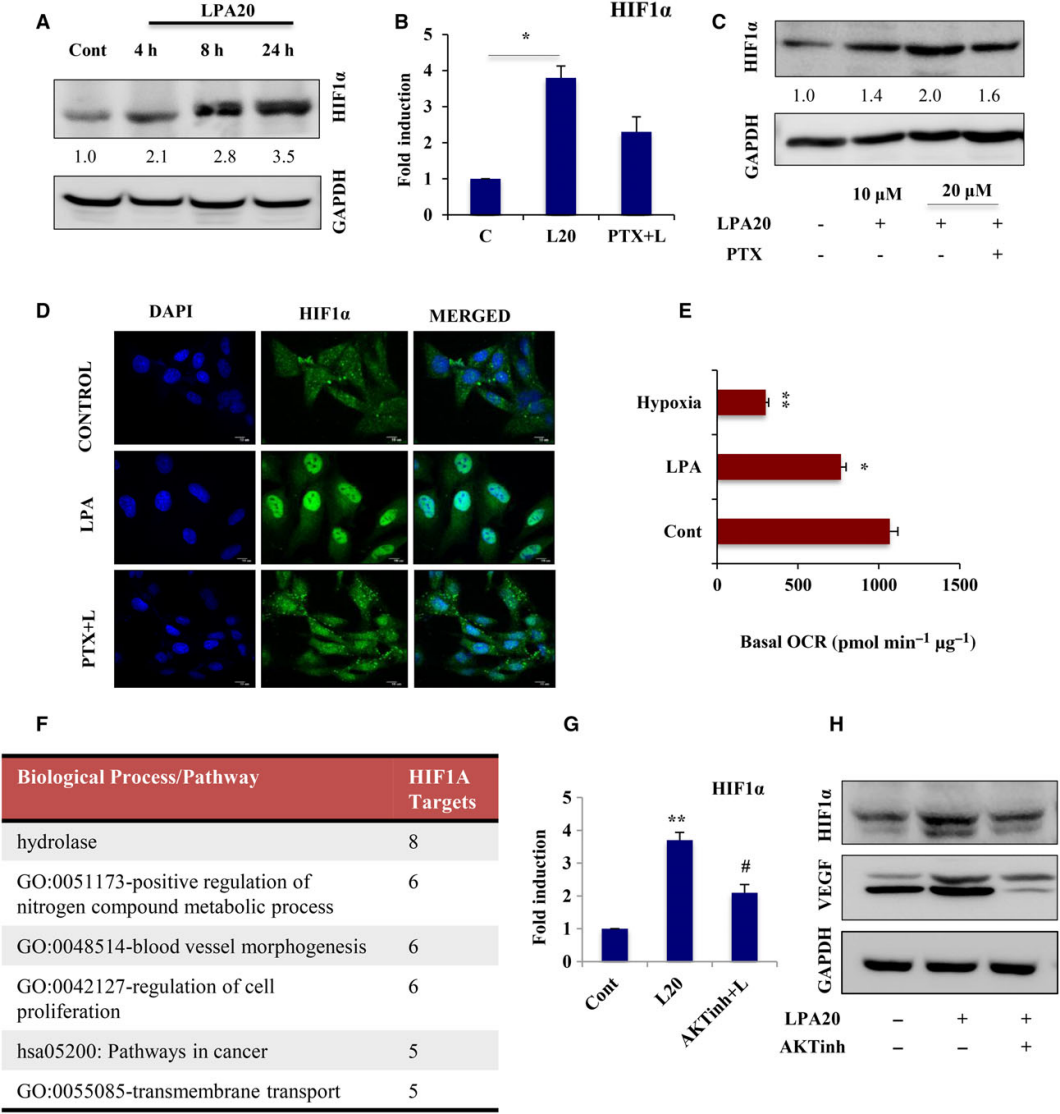
LPA mimics hypoxia through activation of HIF‐1α in ovarian cancer (OC) cells. (A) Western blot analysis of HIF‐1α expression upon treatment with 20 μm LPA in a time‐dependent manner in PA‐1 cells. GAPDH was used as a loading control. HIF‐1α expression was analyzed by (B) quantitative PCR (*p < 0.05) and (C) Western blot in PA‐1 cells treated with LPA in the presence/absence of PTX. In all cases, fold change for the protein expression was calculated using image j software (normalized against GAPDH) and is provided beneath the panel. (D) Nuclear localization of HIF‐1α upon treatment by LPA with or without PTX was observed by confocal imaging. DAPI is used to stain the nucleus (scale bar = 10 μm). (E) OCR in control, LPA‐ and CoCl_2_‐treated PA‐1 cells under basal condition was analyzed using Seahorse extracellular flux analyzer. Representative basal OCR (pmol·min^−1^·μg^−1^) from three independent experiments are plotted; error bars indicate mean ± SEM (*p < 0.05, **p < 0.01). (F) GSEA analysis represented the differentially expressed target genes that gets activated under the control of HIF‐1α transcription factor. (G) Quantitative PCR (**p < 0.01 vs control, #*p* < 0.05 vs LPA treatment) and (H) immunoblot analysis for HIF‐1α expression in cells pretreated with AKT inhibitor for 1 h followed by LPA‐induction. VEGF expression was also observed as a known transcriptional target of HIF‐1α under this condition.

Previous studies have reported that LPA upregulates HIF‐1α in numerous cancer types and we found a time‐dependent increase in HIF‐1α expression in response to LPA (Fig. 7A). LPA‐induced expression and nuclear translocation of HIF‐1α are further reduced in the presence of the LPA receptor inhibitor PTX (Fig. 7B–D). Moreover, we have previously shown that LPA lowers the OCR level (Fig. 1B), which was found to imitate to some extent the condition prevailing in the cells under hypoxia (Fig. 7E). This metabolic plasticity of cancer cells determines a shift towards enhanced glycolytic dependency that is required for maintenance of the invasive fate. LPA‐mediated activation of HIF‐1α was further analyzed through clustering of its differentially expressed targets, which revealed enrichment of key genes associated with angiogenesis, proliferation and nitrogen metabolism (Fig. 7F). Moreover, treatment with AKT inhibitor significantly reduced the expression of LPA‐induced HIF‐1α and one of its known downstream targets, VEGF (Fig. 7G,H), suggesting involvement of this pathway activation in HIF‐1α induction. Together, the results suggest that LPA is an oncolipid that mimics hypoxia‐like conditions in ovarian cancer cells.

### 3.8 HIF‐1α transactivates *ets‐1* that induces LPA‐mediated invasiveness

**Figure 8:**
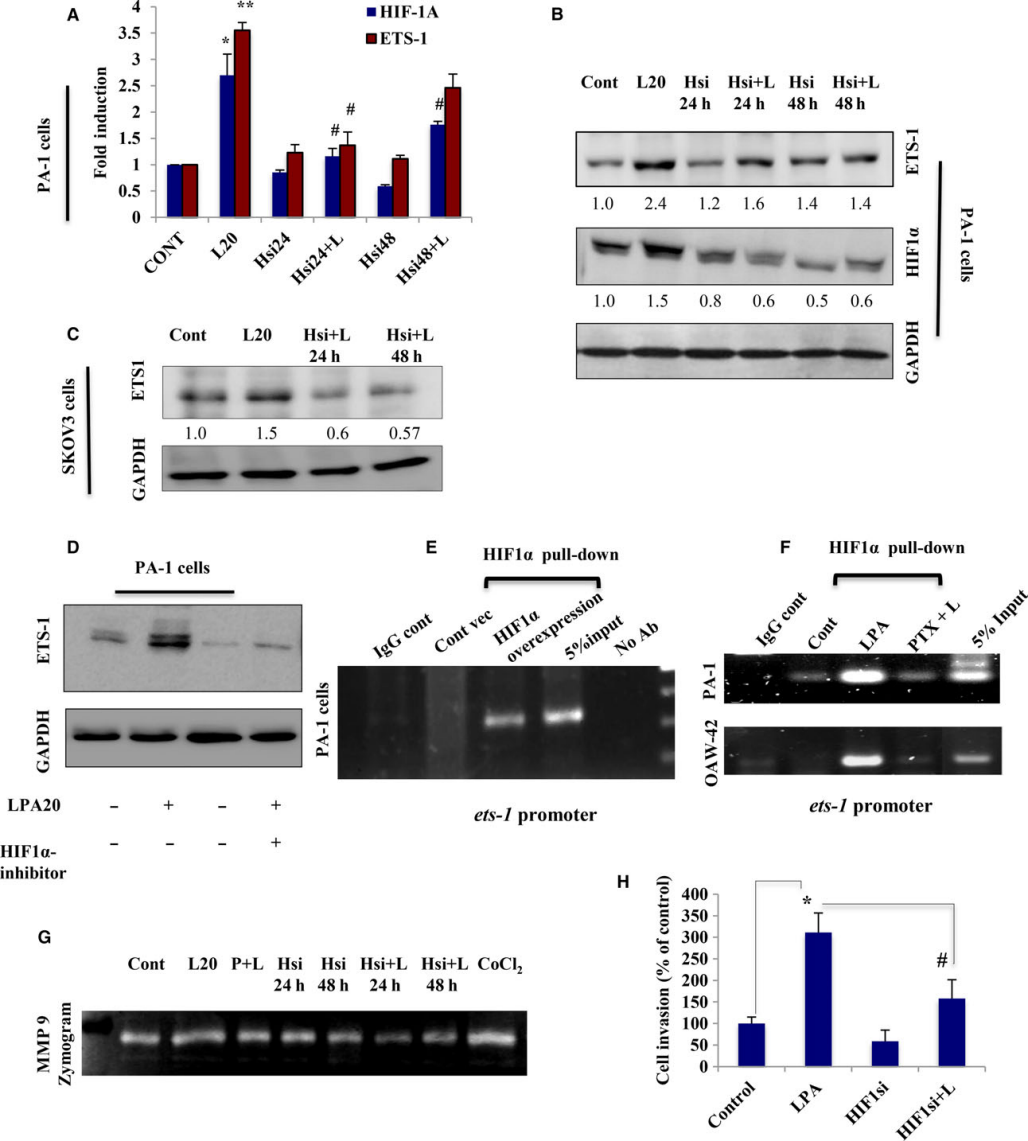
LPA‐induced HIF‐1α transcriptionally upregulates ETS‐1 in ovarian cancer (OC) cells. (A) Quantitative PCR and (B) immunoblot analysis were used to analyze the HIF‐1α and ETS‐1 expression in PA‐1 cells transfected with HIF‐1α‐specific siRNA for 24 and 48 h, followed by LPA treatment (*p < 0.05, **p < 0.01 vs control; #*p* < 0.05 vs LPA treatment). (C) Expression of ETS‐1 was also determined in HIF‐1α‐knockdown SKOV‐3 cells for 24 and 48 h, followed by treatment with LPA. Fold change for the protein expression was calculated using image j software (normalized against GAPDH) and is provided beneath the panel. (D) ETS‐1 expression was further checked in the presence of HIF‐1α inhibitor. (E) ChIP analysis of HIF‐1α towards binding to *ets‐1* promoter region having hypoxia response elements sequences was performed in HIF‐1α‐overexpressed PA‐1 cells and (F) LPA‐treated PA‐1 and OAW‐42 cells. No antibody control and IgG sets were used as negative control. (G) Similar treatments were performed to check the MMP‐9 activity in a gelatin zymogram from the conditioned media. (H) Matrigel Invasion Assay of the HIF‐1α knockdown cells was performed in the absence/presence of LPA and percent cell invasion was plotted (*p < 0.05 vs control; #*p* < 0.05 vs LPA treatment).

To elucidate the existing transcriptional regulation between ETS‐1 and HIF‐1α, we knocked down HIF‐1α and found significant attenuation in LPA‐induced ETS‐1 expression in PA‐1 and OAW‐42 cells (Figs 8A and S6F). Maximal reduction in ETS‐1 protein levels was found at ~ 24 h in PA‐1 (Fig. 8B) and at 48 h in SKOV‐3 and OAW‐42 cells, respectively (Figs 8C and S6G). Treatment with HIF‐1α inhibitor also revealed a decrease in the expression of LPA‐induced ETS‐1 (Fig. 8D). However, no significant change in HIF‐1α expression was observed upon knockdown of ETS‐1 (Fig. S6H). Therefore, HIF‐1α is a critical regulator of ETS‐1 expression under LPA exposure in ovarian cancer.

Further, our *in silico* analysis showed the presence of several hypoxia response elements on the *ets‐1* promoter. To further validate these findings, ChIP for HIF‐1α followed by PCR with primers flanking the hypoxia response elements sequences in *ets‐1* promoter, showed amplification in HIF‐1α‐overexpressed PA‐1 cells compared with control vector‐transfected cells (Fig. 8E). HIF‐1α ChIP–PCR further confirms binding of HIF‐1α to the *ets‐1* promoter in LPA‐stimulated PA‐1 and OAW‐42 cells, where treatment with its receptor inhibitor was found to attenuate the promoter binding (Fig. 8F). The functional significance of this interaction was investigated by assessing the effect of HIF‐1α knockdown on invasive phenomenon of ovarian cancer cells. HIF‐1α silencing results in reduced MMP‐9 levels, along with a decreased rate of invasion (Fig. 8G,H). Collectively, it can be suggested that transcriptional induction of ETS‐1 is directly regulated by HIF‐1α in ovarian cancer cells.

### 3.9 LPA treatment upregulates ETS‐1 and HIF‐1α in *in vivo* syngeneic mice model

**Figure 9:**
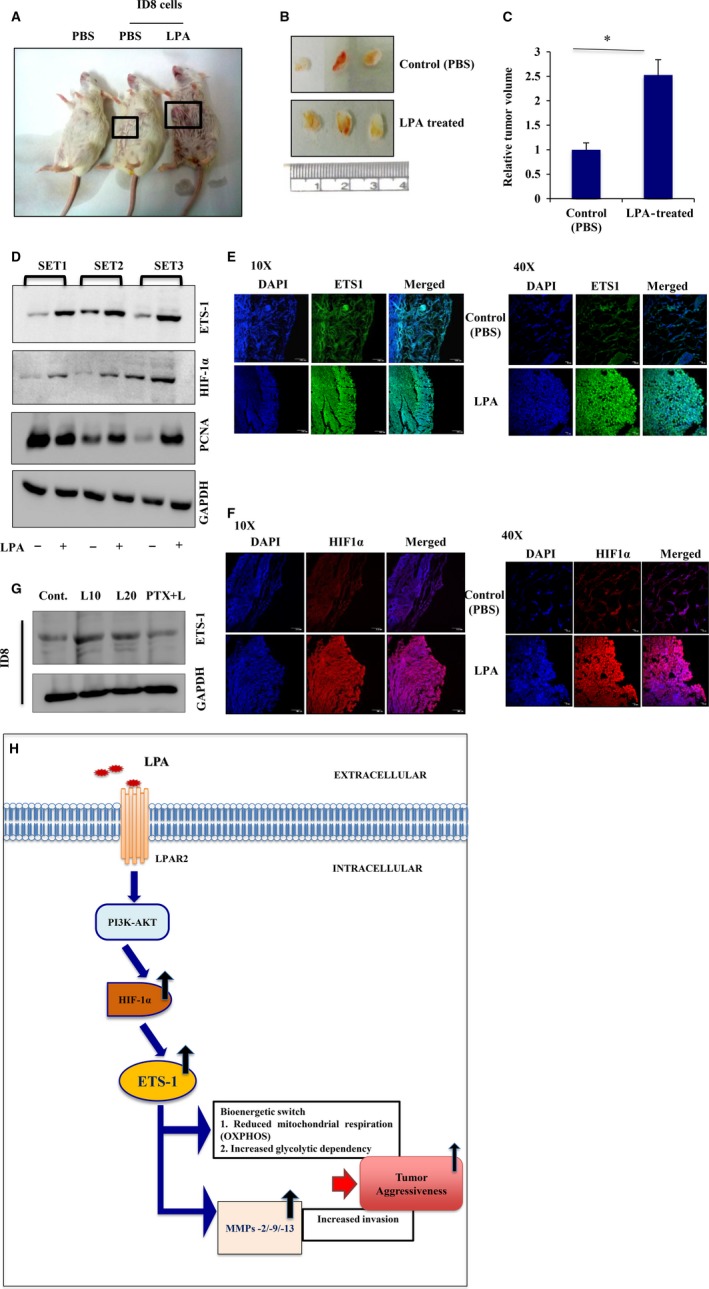
LPA is functionally involved in the upregulation of ETS‐1 *in vivo*. (A) Representative images of tumors formed in BALB/c mice injected with either PBS alone or ID8 cells with PBS or LPA. (B) Effect of LPA treatment on tumor growth in BALB/c mice. (C) Tumor volume was measured and represented as fold change (*p < 0.05). (D) Western blotting was performed with three sets of tumor samples as indicated, for ETS‐1 and HIF‐1α expression analysis. Proliferating cell nuclear antigen expression was observed in these samples as a marker of proliferation and GAPDH was used as a loading control. (E) ETS‐1 and (F) HIF‐1α expression levels were analyzed in these tissue sections by fluorescence‐immunohistochemistry using confocal microscopy (scale bar = 10 and 100 μm). (G) ETS‐1 expression was analyzed in ID8 cells upon treatment with LPA in the presence and absence of its receptor inhibitor to validate the *in vivo* data. (H) Schematic representation of LPA‐induced ovarian cancer aggressiveness. LPA, through LPAR2‐specific AKT signaling, activates HIF‐1α and subsequently ETS‐1 leading to reduced mitochondrial respiration and increased glycolysis, as well as cell‐type‐specific proteolytic enzyme expression, thereby specifying a sustained invasive potential for the cancer cells.

To verify the physiological relevance of our *in vitro* findings, we extended our investigation in the syngeneic mice model with ID8 mouse ovarian cancer cells. The primary tumor produced from injected ID8 cells administered with LPA, after 20 days, had a much larger volume (~ 2.5‐fold) than that from mice injected with only ID8 cells (Fig. 9A–C). A significant increase in the expression of ETS‐1 and HIF‐1α was found in the tumor sample from LPA‐treated of mice (Fig. 9D). Proliferating cell nuclear antigen, a proliferation marker, also showed enhanced expression in the LPA‐treated group. Furthermore, immunostaining of tissue sections from LPA‐injected mice showed enhanced expression of both ETS‐1 and HIF‐1α (Fig. 9E,F) on confocal microscopy. The effect of LPA on ID8 cells was verified by observing increased expression of ETS‐1 (Fig. 9G). In addition, a proliferation assay was performed to revalidate the results *in vitro* upon exposure to LPA (Fig. S6I). Hence, our *in vivo* data further confirm the involvement of ETS‐1 and HIF‐1α in LPA‐induced tumorigenesis.

## 4 Discussion

Metabolic adaptations of the tumor cells during cancer progression determine their metastatic potency (Hanahan and Weinberg, 2011). Chemical cues, activation of oncogenes and inactivation of tumor suppressor genes can all have a profound influence on tumor cell phenotypes (Jiang *et al*., 2015). Reprogramming of energy metabolism towards enhanced aerobic glycolysis is a core hallmark of many cancers (Langbein *et al*., 2008; Ramanathan *et al*., 2005). Such increased glycolytic dependency of cancer cells plays a key role in protecting them from the varied stresses that generate intracellular reactive oxygen species (Brand and Hermfisse, 1997). Thereby, glycolysis acts as a critical driver of tumorigenicity; however, the mechanisms leading to repression of mitochondrial oxidative phosphorylation need detailed elucidation.

Elevated levels of the metabolite, LPA, were found in the ascites and plasma of early‐stage ovarian cancer patients, and thus LPA is regarded as a potential biomarker for ovarian cancer (Xu *et al*., 1998). This enrichment of LPA in the tumor microenvironment might be an important cause of further aggravation of ovarian cancer and future studies should focus on determining the in‐depth regulation of its excess production and the mechanisms that lead to advancement of ovarian cancer, in suitable model systems. Accumulated evidence suggests the role of LPA in promoting metastasis in various solid tumors (Fan *et al*., 2015; Fishman *et al*., 2001; Leve *et al*., 2015; Mills and Moolenaar, 2003; Park *et al*., 2011; So *et al*., 2004), through the activation of signaling cascades, mainly p38MAPK and Rho/ROCK (Bian *et al*., 2006; Jeong *et al*., 2012; Sautin *et al*., 2001). Because the invasion and metabolic adaptations of cancer cells are strongly associated, this study aimed to establish the contribution of this oncolipid to altered metabolic adaptations supporting the invasive potential of ovarian cancer cells. Therefore, we uncovered the bioenergetic status of ovarian cancer cells that ensues during LPA‐induced ovarian cancer progression. Our report is the first to observe that long‐term exposure to LPA leads to bioenergetic modulation of ovarian cancer cells, which might play a central role in its increased invasive potential compared with non‐transformed epithelial cells. LPA was found to effectively reduce the basal and maximal OCR in ovarian cancer cells, whereas maximal OCR is slightly reduced in non‐transformed cells, thereby indicating a metabolic switch. This was further supported by a significant increase in the glycolytic rate, as well as in lactate levels, along with the enriched glycolytic gene expression profile in ovarian cancer cells. These data predict an enhanced glycolytic adaptation in LPA‐treated ovarian cancer cells, which may be the reason for their increased invasiveness.

The metabolic plasticity of cancer cells on exposure to LPA prompted us to identify the gene‐regulatory network controlling these systemic perturbations. RNA‐Seq analysis revealed the global outlook of the signature genes that determine the invasive fate of the cells on LPA stimulation. Earlier single‐gene studies have suggested the importance of LPA for proliferation/metastasis; however, these studies did not reveal global and comprehensive gene‐regulatory events. Our GSEA analysis established the activation of key oncogenic pathways; among these, we confirm the importance of PtdIns3K–AKT signaling in the enhanced aggressiveness associated with ovarian cancer. Transcriptional reprogramming underlying LPA‐promoted oncogenesis was found to involve a large repertoire of critical transcription factors like MYC, ETS‐1, MEF2A, ERBB2, LIF and SPP1. Based on our findings, regarding the involvement of key MMPs towards the increased invasiveness associated with AKT activation in ovarian cancer cells, we attained the significant transcriptional activation of ETS‐1 oncogene.

Increased expression of ETS‐1 is associated with cancer metastasis (Behrens *et al*., 2001; Dittmer, 2003; Ghosh *et al*., 2012; Nakada *et al*., 1999; Nakayama *et al*., 1996; Span *et al*., 2002), and its forced expression leads to a transformed phenotype, as shown by tumor formation in xenograft mice (Topol *et al*., 1992). We further confirmed that AKT‐induced ETS‐1 is responsible for the increased invasive potential through direct transcriptional regulation of MMP‐2, MMP‐9 and MMP‐13 in a cell type‐specific pattern. We also found Gi‐LPAR2 activation to be significantly responsible for ETS‐1‐mediated ovarian cancer tumorigenesis. Furthermore, current studies revealed the association of ETS‐1 expression with metabolism and oxidative stress in cancer cells (Verschoor *et al*., 2013). ETS‐1 overexpression has also been linked to the upregulation of key enzymes involved in glycolysis and associated feeder pathways (Verschoor *et al*., 2010). Herein, we found that knockdown of ETS‐1 leads to increased oxidative phosphorylation and reduced ECAR, thereby reversing the glycolytic dependency of the LPA‐treated cancer cells. These results offer direct evidence of the glycolytic predisposition observed in LPA‐treated ovarian cancer cells, involving ETS‐1 induction. This further predicts a non‐migratory shift, which supports the fact that inactivation of ETS‐1 can prevent cancer progression.

These observations have proved critical to our understanding of ETS‐1 function in LPA‐induced tumorigenesis, however, we wanted to gain a full understanding of its regulatory mechanism in ovarian cancer. Although studies have highlighted post‐transcriptional regulation of ETS‐1 activity (Ladykowska *et al*., 2002), as well as protein–protein interaction defining its target specificity (Li *et al*., 2000), relatively little is known about the transcriptional regulation ETS‐1 activity in different cancers. Prior studies have suggested induction of ETS‐1 in response to oxidative stress (Wilson *et al*., 2005); implying that the tumor microenvironment may drive increased ETS‐1 expression, which enhances the ability of these cells to promote metastasis. Indeed, we found significant upregulation of ETS‐1 under hypoxia, which is reduced on silencing of HIF‐1α and suggests a strong association between these two factors. Besides, evidence has also shown that hypoxia is not the only condition for HIF‐1α induction. Several growth factors, metabolites or activated oncogenes can induce HIF‐1α under non‐hypoxic conditions in numerous cancers (Fukuda *et al*., 2002; Richard *et al*., 2000; Stiehl *et al*., 2002; Tacchini *et al*., 2001). Additionally, it is well known that LPA leads to enhanced HIF‐1α expression followed by activation of its downstream targets, such as VEGF, which promotes cancer progression (Ha *et al*., 2016; Lee *et al*., 2006; Yang *et al*., 2008). We also found that LPA activation bio‐energetically reorients cancer cells towards a pseudo‐hypoxic condition through HIF‐1α induction. We therefore attempted to identify the regulatory mechanism that might persist between HIF‐1α and ETS‐1, in association with LPA‐induced tumorigenesis. Here, we report the transcriptional induction of ETS‐1 upon direct binding to its promoter through HIF‐1α in both HIF‐1α‐overexpressed cells and LPA‐induced ovarian cancer cells.

Collectively, this work summarizes oncolipid‐mediated aggressiveness in ovarian cancer cells that results from less‐characterized cellular metabolic adaptations. This metabolic reprogramming towards increased glycolysis might play a critical role in the aggressiveness of cancer cells in an LPA‐enriched microenvironment. Thereby, LPA stimulation leads to a condition where HIF‐1α and ETS‐1 together promote the invasive phenomenon through bioenergetic modulation, as well as constitutively activating the MMPs. Because the importance of this oncolipid lies in the fact that it is highly enriched in the tumor microenvironment promoting cancer metastasis, proper modulation of its production as well as function may be useful for the future clinical management of ovarian cancer.

## 5 Conclusions

In conclusion, our study is the first to depict a scenario in which oncolipid (LPA) signaling leads to metabolic plasticity in ovarian cancer cells towards reduced mitochondrial oxidative phosphorylation, as well as increased glycolysis through the pro‐metastatic protein ETS‐1. Thereby, LPA‐induced ETS‐1 was found to maintain the invasive potency of ovarian cancer cells in two key aspects, first, by switching the metabolic dependency of the cancer cells towards increased glycolysis, and second, by regulating the expression of MMPs in a cell‐type specific manner. Further, the complex relationship between the aggressiveness and invasive potential of the cancer cells to the bioenergetic modification, reinforces the crucial role of metabolic plasticity as a driver of metastatic potential. The metabolic dependency on glycolysis from mitochondrial oxidative phosphorylation is promising event in maintaining the survival and invasive potential of the cancer cells, which makes these cells vulnerable to various metabolic inhibitors. Furthermore, detailed insight into the mechanisms involved will open the door to future drug development, in which we propose that specific metabolic inhibitors in combination with other existing anti‐cancer agents may have a promising role in the clinical management of ovarian cancer metastatic progression.

## Authors' contributions

SSR conceived and coordinated the study, and drafted the manuscript. UR designed, performed and analyzed the experiments, and drafted the manuscript. SRC provided technical assistance, performed experiments and corrected the manuscript. MV and KR analyzed and generated the RNA‐Seq analysis data and reviewed the manuscript. SR critically reviewed and partly drafted the manuscript. All authors reviewed the results and approved the final version of the manuscript.

## Supporting information


**Fig. S1**. LPA induces glycolytic transcripts in OC cells.
**Fig. S2**. LPA promotes EMT/invasion/migration in ovarian cells.
**Fig. S3**. PtdIns3K–AKT pathway is crucial for LPA‐mediated response in OC cells.
**Fig. S4**. LPA upregulates the ETS‐1 expression.
**Fig. S5**. LPAR2 is responsible for ETS‐1 expression in OC cells.
**Fig. S6**. ETS‐1 expression is regulated by HIF‐1α in OC cells.
