# Supplementary material for: Gene regulatory networking reveals the molecular cue to lysophosphatidic acid‐induced metabolic adaptations in ovarian cancer cells
Source: Mol Oncol. 2017 Apr 3;11(5):491–516. doi: 10.1002/1878-0261.12046 (PMC5527468; doi:10.1002/1878-0261.12046)
Supplement: Supplementary file 1 — Fig. S1. LPA induces glycolytic transcripts in OC cells. Fig. S2. LPA promotes EMT/invasion/migration in ovarian cells. Fig. S3. PtdIns3K–AKT pathway is crucial for LPA‐mediated response in OC cells. Fig. S4. LPA upregulates the ETS‐1 expression. Fig. S5. LPAR2 is responsible for ETS‐1 expression in OC cells. Fig. S6. ETS‐1 expression is regulated by HIF‐1α in OC cells. [file MOL2-11-491-s001.pdf]

Gene regulatory networking reveals the molecular cue to lysophosphatidic acid-induced metabolic adaptations in ovarian cancer cells

Upasana Ray[1] (upasana.roy.88@gmail.com)

Shreya Roy Chowdhury[1] (shreyaroychowdhury@ymail.com)

Madavan Vasudevan[2] (madavan@bionivid.com)

Kiran Bankar[2] (kiran@bionivid.com)

Susanta Roychoudhury[3] (susantarc@gmail.com)

Sib Sankar Roy[1] (sibsankar@iicb.res.in)*

[1]Cell Biology and Physiology Division, CSIR-Indian Institute of Chemical Biology, Council of Scientific and Industrial Research, 4 Raja S. C. Mullick Road, Kolkata 700032, India.

[2]Bionivid Technology Private Limited, 3rd Floor, 4C-209,4th Cross, Near New Horizon College, Kasturi Nagar, Bangalore-560043, India.

[3]Saroj Gupta Cancer Centre and Research Institute, Mahatma Gandhi Road, Thakurpukur, Kolkata 700063, India.

*Address correspondence to: Dr. Sib Sankar Roy
Cell Biology and Physiology Division, CSIR-Indian Institute of Chemical Biology, Council of Scientific and Industrial Research, 4 Raja S. C. Mullick Road, Kolkata 700032 (India), Tel. + 91-33-2499-5858, Fax + 91-33-2473-5197. Email: sibsankar@iicb.res.in

Supplementary information

Figure Supplementary 1: LPA induces glycolytic transcripts in OC cells. (A) Pie-chart providing quantitative estimation of glycolytic gene expression analysis from the RNA-Seq data. (B) Q-PCR expression validation of HK2 and PKM2 glycolytic genes upon LPA treatment in PA-1 cells (* $p<0.05$).

Figure Supplementary 2: LPA promotes EMT/invasion/migration in ovarian cells. (A) Immunoblot was done to analyze the effect of LPA on E-cad, N-cad and Vim expression in IOSE. (B) Q-PCR analysis was performed to check the expression of *CDH1, CLDN7, CDH2* and *VIM* in LPA-treated IOSE cells (* $p<0.05$). TGF-β was used as a positive inducer of EMT in all the cases. (C) Matrigel invasion images were provided in both the IOSE and PA-1 cells after treatment with LPA in absence and presence of PTX for 22h. (D) Wound healing assay was performed in PA-1 cells after 24h treatment. 0h signifies the time of scratching the cells. The arrows indicate the width of wound and the assay was repeated three times independently. (E) Similar invasion assay was performed in OAW-42 cells. (F) Heatmap showing cluster of differentially expressed tumor invasion effector genes in PA-1 cells through LPA. (G) MMPs-2/-9/-13 mRNA expression was analyzed by Q-PCR after treatment with LPA in absence and presence of PTX (* $p<0.05$, ** $p<0.01$ vs control; # $p<0.05$ vs LPA treatment) in OAW-42 cells. (H) Western blot analysis for the respective MMPs were performed with similar treatments as mentioned. (I) Gelatin zymography assay was performed in OAW-42 cells with similar treatments. (J) Transwell invasion images were provided after the cells were transfected with the indicated siRNAs followed by LPA induction for 22h.

Figure Supplementary 3: PI3K-AKT pathway is crucial for LPA-mediated response in OC cells. (A) GSEA analysis was shown disclosing the key pathways related to cancer progression having statistically significant enrichment ($p\leq0.05$) upon LPA treatment in PA-1 cells. (B) Invasion assay was performed with OAW-42 cells pre-treated by the indicated pathway inhibitors for 1h followed by LPA for 22h (scale bar, 100μm). Percent cell invasion was plotted with cells counted at three independent fields for each well (* $p<0.05$ vs control, # $p<0.05$ vs LPA treatment).

Figure Supplementary 4: LPA up-regulates the ETS-1 expression. (A) The cluster of differentially expressed ETS-family members upon LPA-stimulation were represented by heatmap. (B) The expression of ETS-1 in OAW-42 cells were assessed by QPCR (** $p<0.01$ vs control, # $p<0.05$ vs LPA) and immunoblot after treatment with LPA in presence/absence of PTX. (D) ETS-2 expression was further checked under similar treatments in PA-1 cells. (D) ETS-1 expression was analyzed under chronic exposure to LPA in non-cancer IOSE cells (* $p<0.05$). (E) Expression analysis of two glycolytic genes LDHA and PKM2 were performed upon knockdown of ETS-1 in LPA-treated PA-1 OC cells (* $p<0.05$ vs control, # $p<0.05$ vs LPA treatment). (F) ChIP-PCR with PA-1 cells showed the amplification of the (i) *MMP-9* and (ii) *MMP-2* promoter from the chromatin input and ETS1-IP DNA as indicated. Amplification was not observed when performed with no antibody control sets.

Figure Supplementary 5: LPAR2 is responsible for ETS-1 expression in OC cells. (A) Expression analysis by PCR of the presence of three LPA-specific receptors (LPAR1/2/3) in

the PA-1 and SKOV-3 cell lines was provided. 18sRNA was used as a positive control. (B) OAW-42 and (C) SKOV-3 cells were transfected with the LPA receptor-specific siRNAs and stimulated with 20μM LPA for 24h, followed by western analysis of ETS-1. GAPDH was used as a loading control. Densities of the respective bands were calculated by Image J software (normalized against GAPDH) and represented as 'fold change' beneath the panel.

Figure Supplementary 6: ETS-1 expression is regulated by HIF-1α in OC cells. (A) HIF-1α expression was analyzed by treatment of PA-1 cells with $CoCl_2$ at a conc. of 100μM and 200μM. ETS-1 levels were quantified by (B) Q-PCR and (C) western blot in presence of 100μM $CoCl_2$. (D) ETS-1 expression levels were assessed in HIF-1α knockdown cells exposed to hypoxia by $CoCl_2$ treatment through Q-PCR and (E) immunoblot detection. (F) Q-PCR and (G) immunoblot analysis were performed to analyze ETS-1 expression in OAW-42 cells transfected with HIF1α-specific siRNA for 24h/48h, followed by LPA treatment (*p<0.05 vs control; #p<0.05 vs LPA treatment). (H) Expression status of HIF-1α in ETS-1 knockdown cells was performed for 24h/48h. GAPDH was used as loading control. (I) Cell proliferation assay in response to LPA was obtained by the MTT method. Percent cell viability was plotted compared to the control (*p<0.05 vs control, #p<0.05 vs LPA).

A

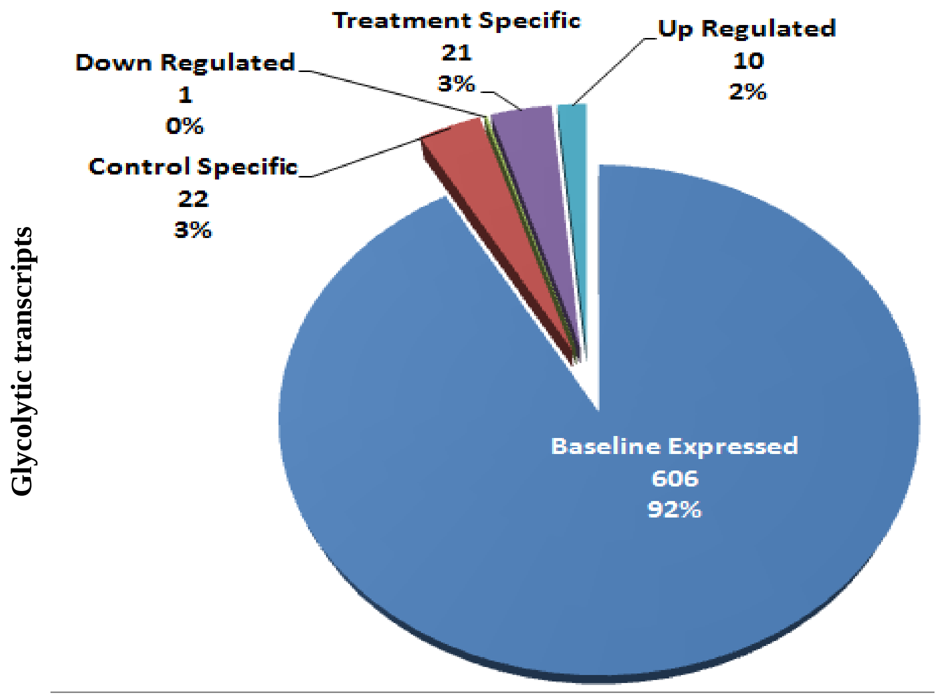

B

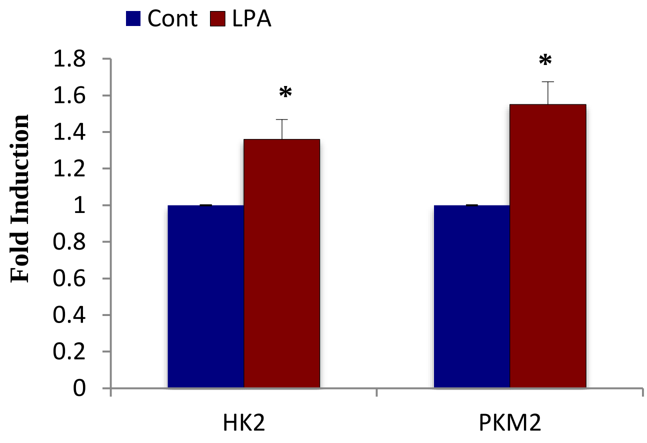

Supplementary 1

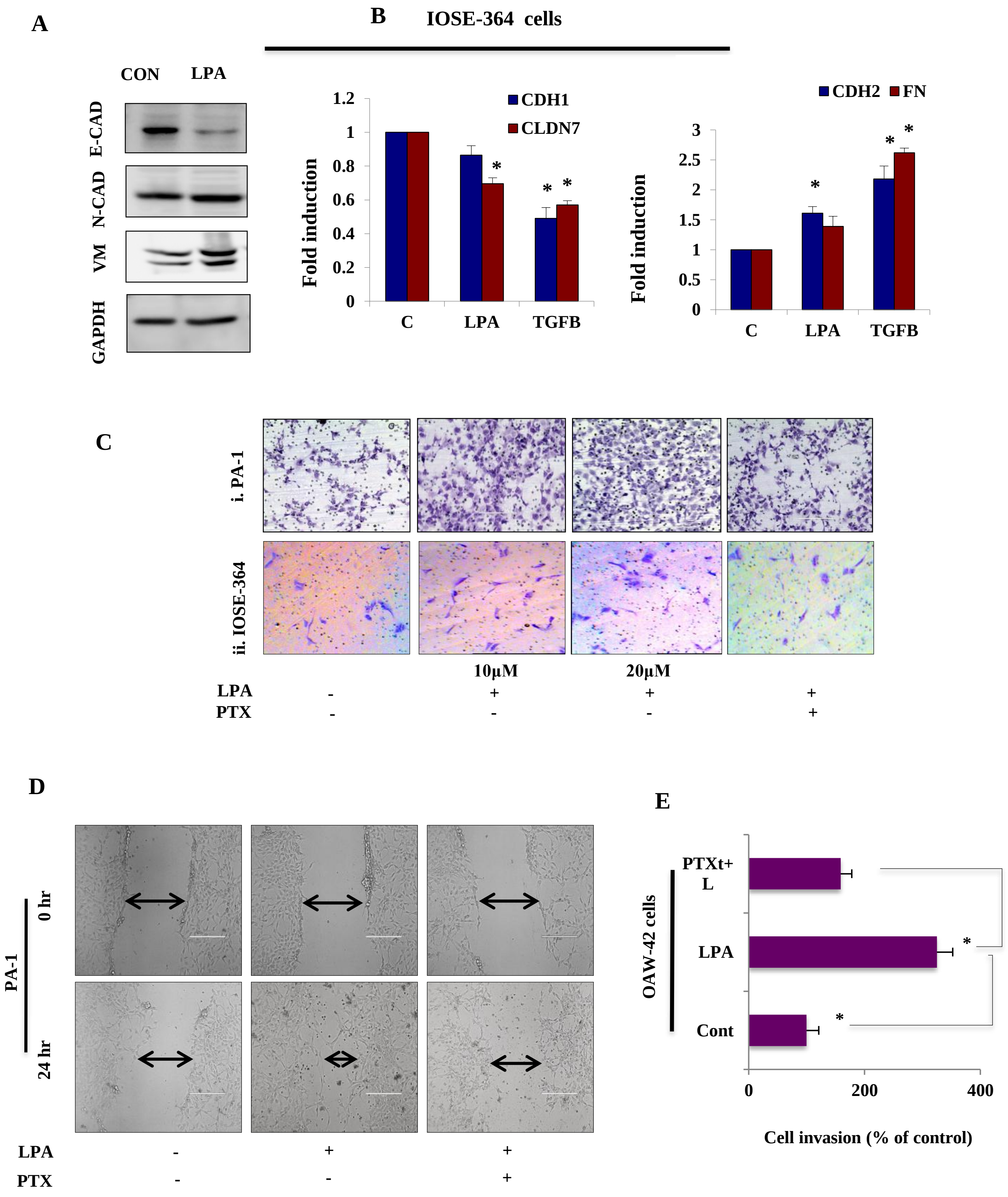

Supplementary 2 cont.

F

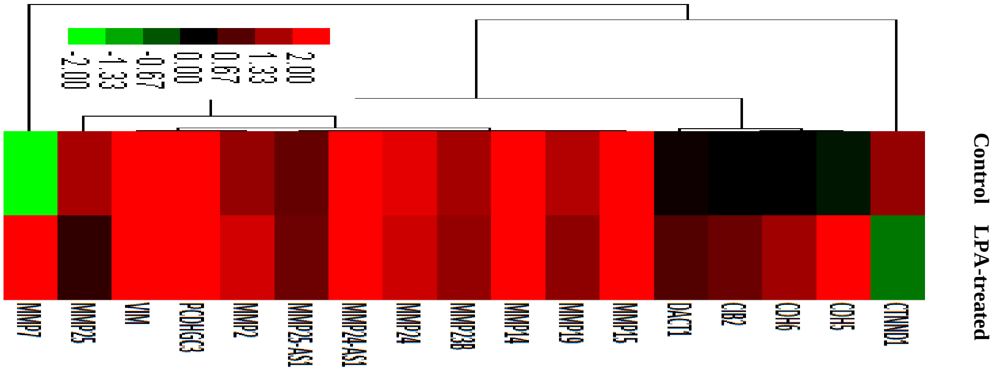

G

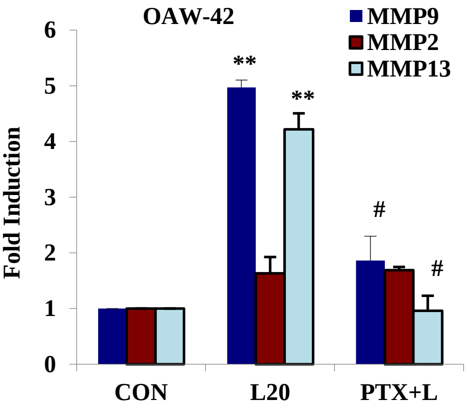

H

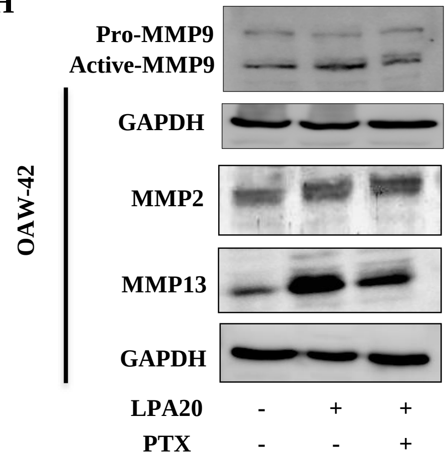

I

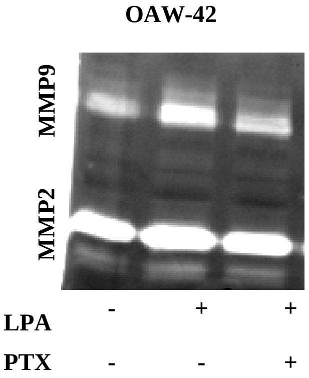

J

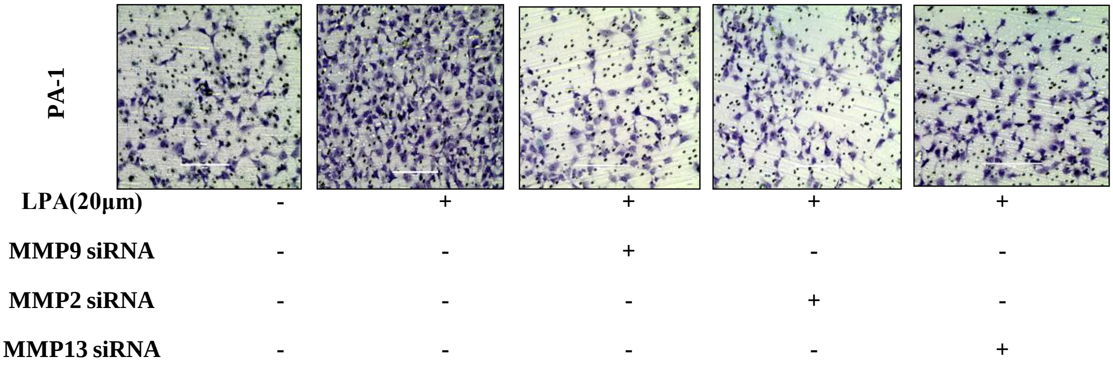

Supplementary 2

A

Pathways	Up Regulated	Down Regulated
EMT_Pathway	18	0
hsa04510:Focal adhesion	20	1
hsa04010:MAPK signaling pathway	16	2
REACT_604:Hemostasis	15	1
hsa05200:Pathways in cancer	14	4
hsa04310:Wnt signaling pathway	9	4
hsa04512:ECM-receptor interaction	9	0
hsa05410:Hypertrophic cardiomyopathy (HCM)	8	0
REACT_13552:Integrin cell surface interactions	8	0
hsa05210:Colorectal cancer	7	2
hsa05222:Small cell lung cancer	7	1
hsa05215:Prostate cancer	6	2
BBID_114.Genomic_reformatting_Brain_Ischemia	5	0
hsa04151: PI3K-Akt signaling pathway	25	24

B

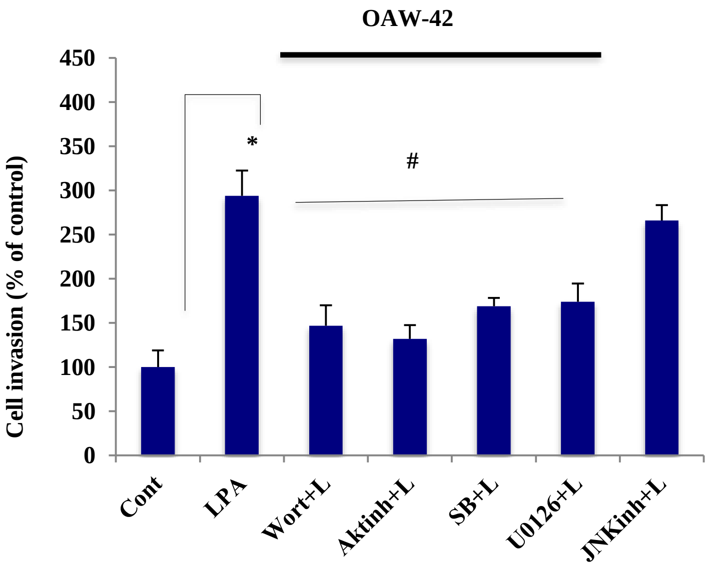

Supplementary 3

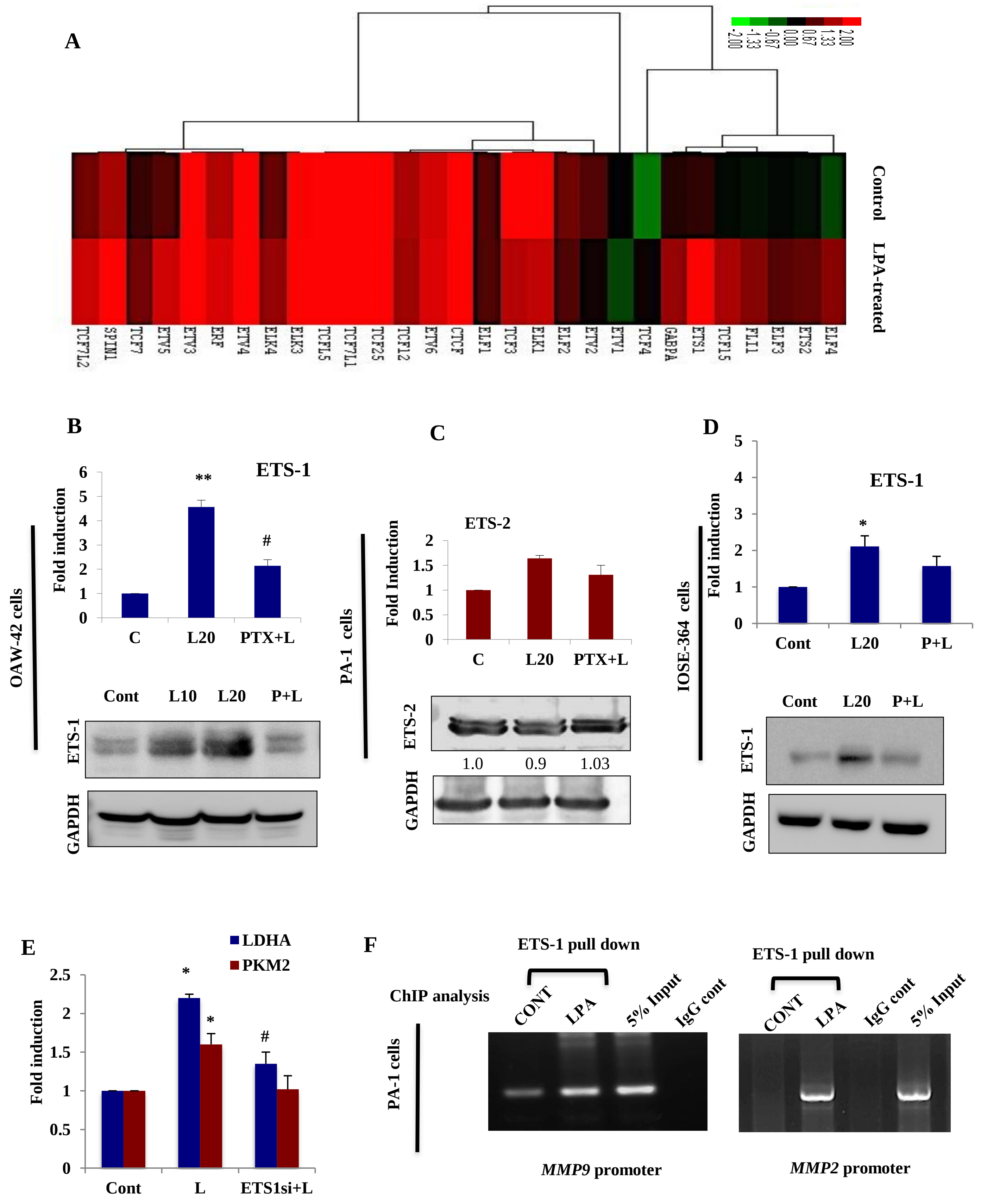

Supplementary 4

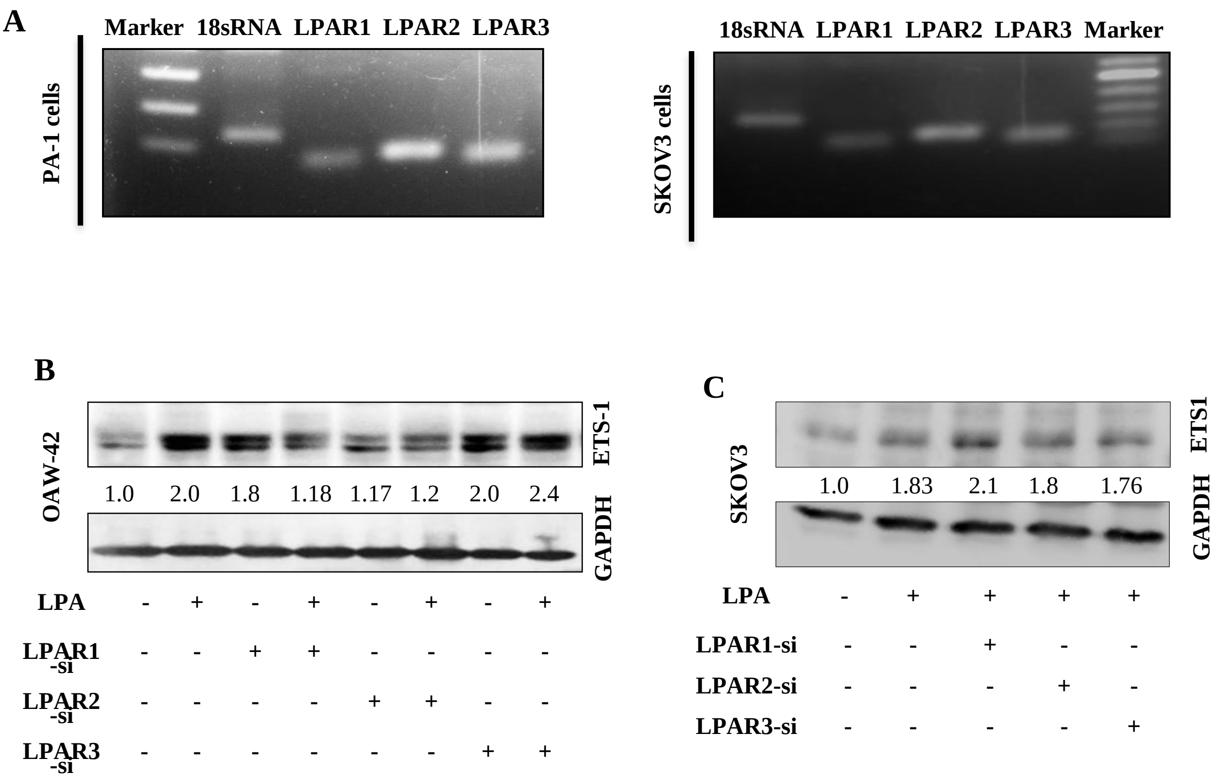

Supplementary 5

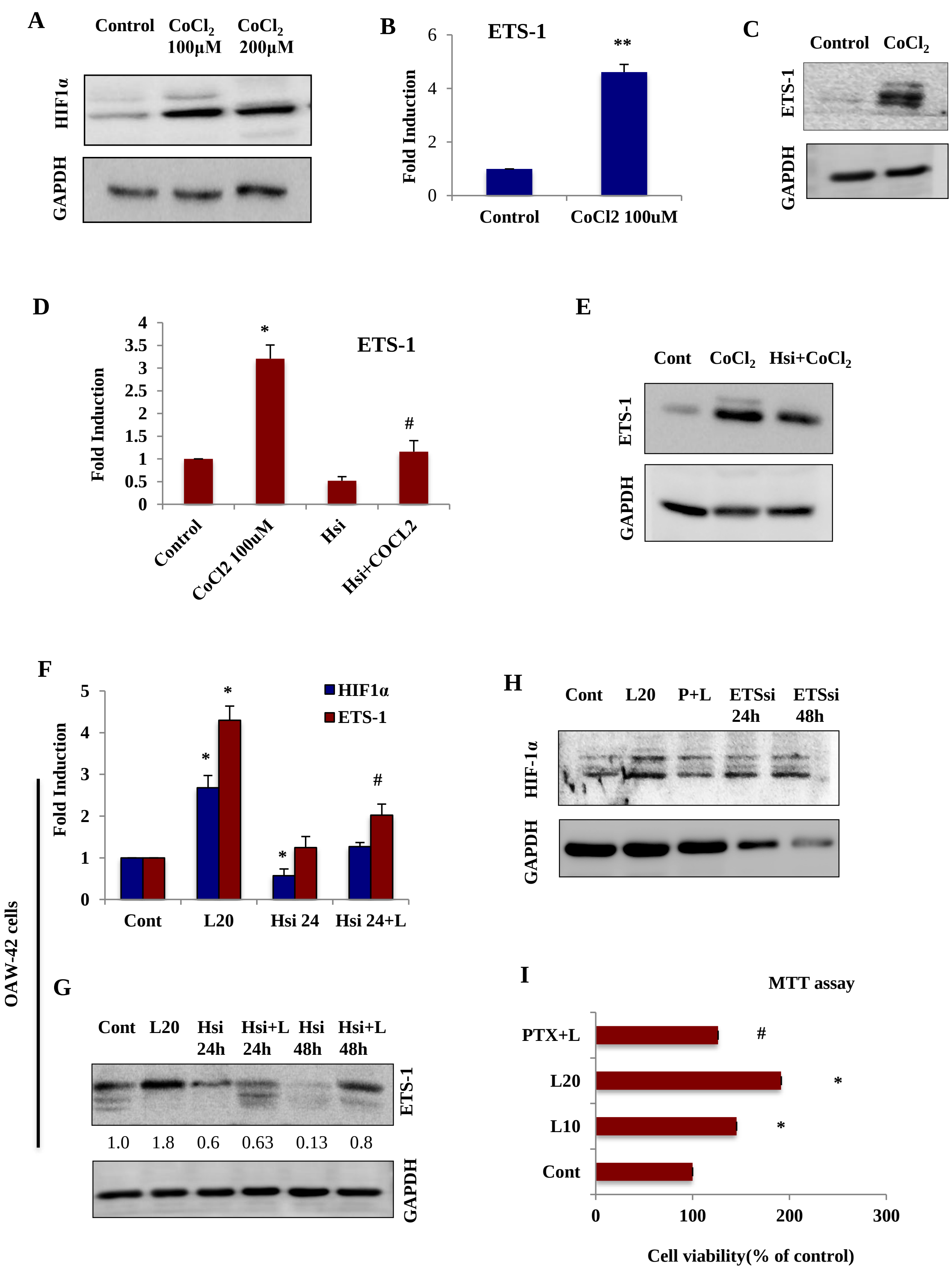

Supplementary 6
